# Pentose degradation in archaea: *Halorhabdus* species degrade D-xylose, L-arabinose and D-ribose via bacterial-type pathways

**DOI:** 10.1007/s00792-020-01192-y

**Published:** 2020-08-05

**Authors:** Jan-Moritz Sutter, Ulrike Johnsen, Andreas Reinhardt, Peter Schönheit

**Affiliations:** Institut für Allgemeine Mikrobiologie, Christian-Albrechts-Universität Kiel, Am Botanischen Garten 1-9, 24118 Kiel, Germany

**Keywords:** *Halorhabdus utahensis*, d-ribose, d-xylose and l-arabinose, Archaea, Xylose isomerase, Ribokinase, Lateral gene transfer

## Abstract

The degradation of the pentoses d-xylose, l-arabinose and d-ribose in the domain of archaea, in *Haloferax volcanii* and in *Haloarcula* and *Sulfolobus* species, has been shown to proceed via oxidative pathways to generate α-ketoglutarate. Here, we report that the haloarchaeal *Halorhabdus* species utilize the bacterial-type non-oxidative degradation pathways for pentoses generating xylulose-5-phosphate. The genes of these pathways are each clustered and were constitutively expressed. Selected enzymes involved in d-xylose degradation, xylose isomerase and xylulokinase, and those involved in l-arabinose degradation, arabinose isomerase and ribulokinase, were characterized. Further, d-ribose degradation in *Halorhabdus* species involves ribokinase, ribose-5-phosphate isomerase and d-ribulose-5-phosphate-3-epimerase. Ribokinase of *Halorhabdus tiamatea* and ribose-5-phosphate isomerase of *Halorhabdus utahensis* were characterized. This is the first report of pentose degradation via the bacterial-type pathways in archaea, in *Halorhabdus* species that likely acquired these pathways from bacteria. The utilization of bacterial-type pathways of pentose degradation rather than the archaeal oxidative pathways generating α-ketoglutarate might be explained by an incomplete gluconeogenesis in *Halorhabdus* species preventing the utilization of α-ketoglutarate in the anabolism.

**Electronic supplementary material:**

The online version of this article (10.1007/s00792-020-01192-y) contains supplementary material, which is available to authorized users.

## Introduction

**Fig. 1:**
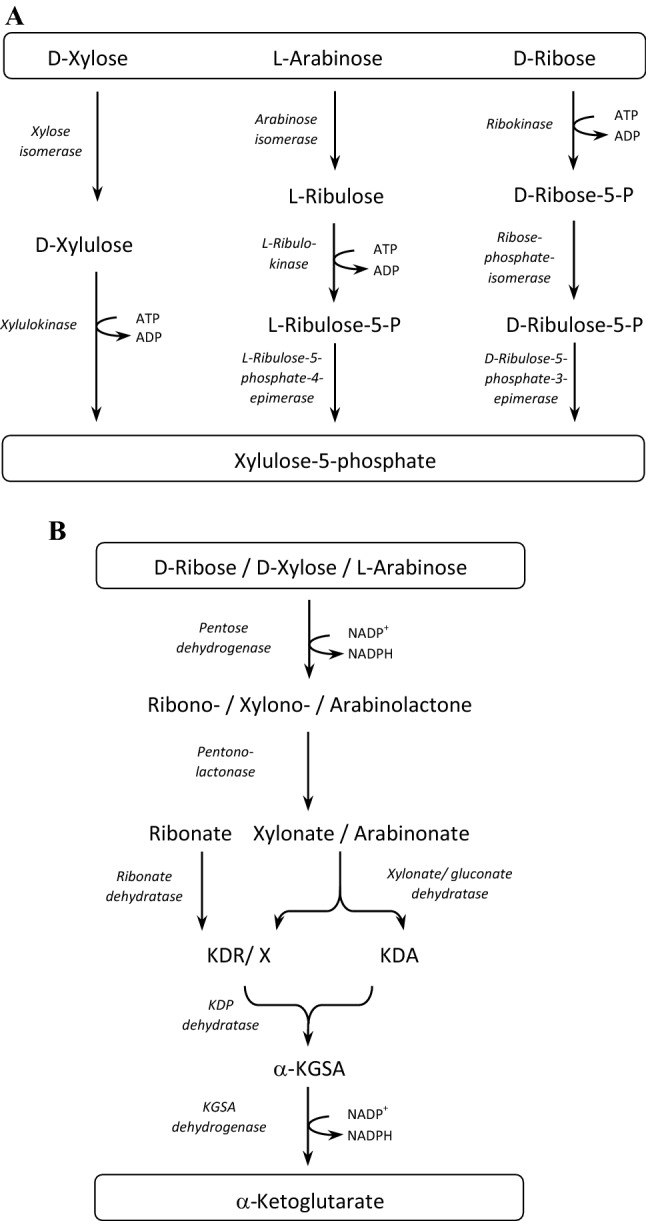
Proposed pathways of pentose degradation in bacteria and archaea.** a** Non-oxidative degradation pathways of D-xylose, L-arabinose and D-ribose to xylulose-5-phosphate operative in most bacteria. **b** Oxidative degradation pathway of D-ribose, D-xylose and L-arabinose to α-ketoglutarate in the *Haloarcula* species *H. marismortui* and *H. hispanica*. Abbreviations: KDR, 2-keto-3-deoxyribonate; KDX, 2-keto-3-deoxyxylonate; KDA, 2-keto-3-deoxyarabinonate; α-KGSA, α-ketoglutarate semialdehyde; KDP dehydratase, 2-keto-3-deoxypentanonate dehydratase

The pentoses d-xylose, l-arabinose and d-ribose are abundant in nature being part of hemicellulose material of plants and as component of ribonucleotides. Thus, these pentoses are common growth substrates of many microorganisms. The pathways of pentose degradation have been well studied in bacteria and fungi. In most bacteria d-xylose, l-arabinose and d-ribose are non-oxidatively degraded to xylulose-5-phosphate, an intermediate of the pentose phosphate pathway, involving specific sugar kinases, isomerases and epimerases (Fig. 1a). The degradation of D-xylose involves xylose isomerase and xylulokinase, and of l-arabinose the enzymes arabinose isomerase, ribulokinase and l-ribulose-5-phosphate-4-epimerase. Further, d-ribose conversion to xylulose-5-phosphate is catalyzed by ribokinase, ribose-5-phosphate isomerase and d-ribulose-5-phosphate-3-epimerase (van de Werken et al. 2008).

In the domain of archaea, the degradation of d-xylose, l- and d-arabinose were studied in detail in the haloarchaea *Haloferax volcanii*, the *Haloarcula* species *H. marismortui* and *H. hispanica*, and in the thermoacidophilic *Sulfolobus* species *S. solfataricus* and *S. acidocaldarius*. In *H. volcanii* d-xylose and l-arabinose are degraded to α-ketoglutarate via an oxidative pathway involving specific dehydrogenases for d-xylose and l-arabinose oxidation and promiscuous pentonolactonase and pentanonate dehydratase. Further, α-ketoglutarate is formed by a 2-keto-3-deoxypentanonate dehydratase and an α-ketoglutarate semialdehyde dehydrogenase (Johnsen et al. 2009, 2013; Sutter et al. 2017). The degradation of d-ribose in archaea has been elucidated in *Haloarcula* species, which unlike *H. volcanii* utilize d-ribose. It was found that *Haloarcula* species evolved a novel oxidative pathway of pentose degradation that allows the degradation of d-ribose, in addition to d-xylose and l-arabinose (Johnsen and Schönheit 2004; Johnsen et al. 2020). This degradation pathway involves novel promiscuous enzymes, pentose dehydrogenase and pentonolactonase, that show an expanded substrate specificity for d-ribose and ribonolactone, respectively (Fig. 1b). Further, a novel highly specific dehydratase for ribonate has been identified forming a novel family within the enolase superfamily. The final conversion of 2-keto-3-deoxyribonate to α-ketoglutarate proceeds via 2-keto-3-deoxypentanonate dehydratase and α-ketoglutarate semialdehyde dehydrogenase that are homologous to the respective *H. volcanii* enzymes. In *Sulfolobus* species d-xylose, l- and d-arabinose are oxidatively degraded to α-ketoglutarate and/or malate (Brouns et al. 2006; Nunn et al. 2010; Wagner et al. 2018).

Based on bioinformatic analyses of ten haloarchaeal genomes (Anderson et al. 2011) it has been reported that *Halorhabdus utahensis*—unlike other haloarchaea—contains classical bacterial-like genes of d-xylose and l-arabinose degradation to xylulose-5-phosphate. So far, transcriptional analyses of these genes during growth on pentoses and the catalytic properties of the encoded enzymes have not been analyzed.

Here, we report transcription of selected genes of d-xylose and l-arabinose degradation in *H. utahensis* and the characterization of their encoded enzymes. Further, we found that d-ribose is degraded in *H. utahensis* and *Halorhabdus tiamatea* to xylulose-5-phosphate by the bacterial pathway involving the enzymes ribokinase, ribose-5-phosphate isomerase and d-ribulose-5-phosphate-3-epimerase. Together, the data indicate that in *Halorhabdus* species pentoses are degraded by the classical—non-oxidative—degradation pathways found in most bacteria. Thus, the genus *Halorhabdus* differs from other closely related haloarchaea that degrade pentoses via oxidative pathways. It is proposed that *Halorhabdus* species acquired their pentose catabolic pathways from bacteria via lateral gene transfer.

## Materials and methods

### Growth of *Halorhabdus utahensis*

*Halorhabdus utahensis* (DSM12940) was grown under aerobic conditions at 37 °C on a modified version of the DSMZ medium number 927; the Tris-buffer was exchanged by 3-morpholinopropane-1-sulfonic acid buffer (0.1 M). d-Xylose, d-ribose, l-arabinose or d-glucose (each 15 mM) were used as carbon and energy sources. Growth was followed by measuring the optical density at 600 nm; during growth, samples were taken for measuring the substrate consumption using the Orcinol assay (Johnsen and Schönheit 2004).

### Purification of ribokinase activity from D-ribose grown *H. utahensis*

Cell extract was prepared in 100 mM Tris–HCl (pH 8.0) containing 2 M ammonium sulfate. After application on a Phenyl-Sepharose column, protein was eluted by decreasing the concentration of ammonium sulfate. Fractions containing ribokinase activity (measured with 20 mM d-ribose and 2.5 mM ATP) were further purified by size exclusion chromatography using a Superdex 200 column and 50 mM Tris–HCl (pH 7.5) containing 2 M potassium chloride. Fractions showing ribokinase activity were applied on a Q-Sepharose column in 50 mM Tris–HCl (pH 8.8) containing 0.15 M NaCl and 50 mM MgCl_2_. Protein was eluted by increasing the concentration of NaCl up to 1 M. At this stage, the protein was partially pure as analyzed by SDS-PAGE, and was used for MALDI-TOF analysis.

### Overexpression and purification of recombinant enzymes

Genes were amplified from genomic DNA of *H. utahensis* and each PCR product was cloned into pTA963 using the cloning strategy as described (Allers et al. 2010) (Supplemental Table S1). *H. volcanii* H1209 was transformed with the respective plasmids and expression of each gene was performed in complex medium at 42 °C (Allers et al. 2010); expression was induced by the addition of 2 mM tryptophan. After 18 h of further growth, cells were harvested by centrifugation. Cell pellets were suspended in 50 mM Tris–HCl (pH 8.2) containing 1.5 M KCl, 50 mM MgCl_2_ and 5 mM imidazole and disruption was performed by passing the cells through a French pressure cell followed by a centrifugation step. The supernatants were applied onto a nickel–nitrilotriacetic acid (Ni–NTA) column and specific elution of proteins was performed with 100 mM imidazole. Further purification was performed by size-exclusion chromatography on a Superdex HiLoad 200 column in 50 mM Tris–HCl (pH 7.5) containing 1.5 or 2 M KCl. At this stage most of the proteins were essentially pure. Xylulokinase was further purified on a Phenyl-Sepharose column equilibrated in 50 mM Tris–HCl (pH 8.0) containing 2 M ammonium sulfate. Elution of the protein was performed by decreasing the concentration of ammonium sulfate.

### Determination of native molecular masses of recombinant enzymes

Size-exclusion chromatography was performed with a flow rate of 1 ml/min using a Superdex 200 HiLoad column (1.6 by 60 cm) (GE Healthcare, Freiburg, Germany). For calibration of the column, the HMW and LMW kits (GE Healthcare) were used. For the calculation of the oligomeric structures of the enzymes, the calculated molecular masses of subunits were used. The subunit sizes measured with SDS-PAGEs were higher than the calculated masses due to the acidic nature of extremely halophilic proteins (Pickl et al. 2014).

### Characterization of recombinant enzymes

Xylose isomerase was measured at 37 °C in 0.1 M Tris–HCl (pH 8.0) containing 2 M KCl, 20 mM MgCl_2_, 1 mM CoCl_2_ and 100 mM d-xylose. d-Glucose and l-arabinose were tested as alternative substrates. During incubation (0–30 min), aliquots were taken and the reaction was stopped by the addition of trichloroacetic acid to a final concentration of 10%. After centrifugation product formation was quantified by the cysteine-carbazole method (Horecker 1988).

Xylulokinase was measured at 37 °C in 0.1 M Tris–HCl (pH 8.0) containing 2 M KCl, 10 mM MgCl_2_, 0.3 mM NADH, 1 mM phosphoenolpyruvate, 12.5 mM ATP, 10 mM D-xylulose, 6 U pyruvate kinase and 9 U lactate dehydrogenase.

Arabinose isomerase was measured at 37 °C in 0.1 M Tris–HCl (pH 8.0) containing 2 M KCl, 20 mM MgCl_2_, 1 mM CoCl_2_ and 50 mM L-arabinose. During incubation (0–20 min) aliquots were taken and L-ribulose was quantified by the cysteine-carbazole method.

Ribulokinase was measured at 37 °C in 0.1 M Tris–HCl (pH 8.0) containing 1.5 M KCl, 50 mM MgCl_2_, 0.3 mM NADH, 1 mM phosphoenolpyruvate, 10 mM ATP, 1 mM L-ribulose, 6 units pyruvate kinase and 9 units lactate dehydrogenase.

Ribokinase was measured at 42 °C in 0.1 M bis–tris (pH 7.5) containing 1.5 M KCl, 0.3 mM NADH, 2.5 mM phosphoenolpyruvate, 10 mM MgCl_2_, 5 mM ATP, 5 mM D-ribose, 1 U pyruvate kinase and 5 U lactate dehydrogenase.

Ribose-5-phosphate isomerase was measured at 37 °C in 0.1 M Tris–HCl (pH 8.0) containing 2 M KCl, 20 mM MgCl_2_ and 80 mM D-ribose-5-phosphate. Alternative substrates were tested at 10 mM and 100 mM. During incubation (0–10 min), aliquots were taken and activity was detected by the cysteine-carbazole method.

Enzyme activities of the oxidative pentose degradation pathways, i.e., xylose dehydrogenase, arabinose dehydrogenase, ribonate dehydratase and α-ketoglutarate dehydrogenase, were measured in cell extracts according (Johnsen et al. 2009, 2020, 2013).

### Transcriptional analyses

RNA was prepared from exponentially grown cells of *H. utahensis* (optical density at 600 nm of about 0.5) as described (Johnsen et al. 2013). Northern blot analyses were performed with 2 to 8 µg RNA (Pickl et al. 2012). Probes were generated by PCR using the PCR digoxigenin (DIG) probe synthesis kit (Roche Diagnostics, Mannheim, Germany) (primers are summarized in Supplemental Table S1). Sizes of transcripts were calculated with the RiboRuler high-range RNA ladder (Thermo Fisher Scientific, Schwerte, Germany).

### Sequence and phylogenetic analyses

BlastP analyses were performed using the RefSeq database at NCBI (O'Leary et al. 2016). Sequence alignments were generated with ClustalX 2.1 using default parameters and are provided with ESPRIPT (Larkin et al. 2007; Robert and Gouet 2014). Secondary structure elements of xylose isomerase and ribokinase of *Halorhabdus* species were predicted using the PSIPRED server (Buchan et al. 2013). Phylogenetic trees are based upon a multiple-amino-acid sequence alignment that was generated with ClustalX (Larkin et al. 2007). Numbers at the nodes are bootstrapping values according to neighbor joining (NJ).

## Results and discussion

Genome analyses indicate that *Halorhabdus utahensis* unlike other haloarchaea contains genes encoding putative enzymes of the classical pathways of d-xylose and l-arabinose degradation reported for most bacteria (Anderson et al. 2011)*.* We performed transcript analyses of selected genes of the bacterial pathways and analyzed the molecular and catalytic properties of the encoded enzymes. Also, D-ribose degradation in *H. utahensis* and *Halorhabdus tiamatea* and the enzymes involved were studied revealing the operation of the bacterial-type pathway of D-ribose degradation.

### d-xylose degradation to xylulose-5-phosphate in *H. utahensis*

**Fig. 2:**
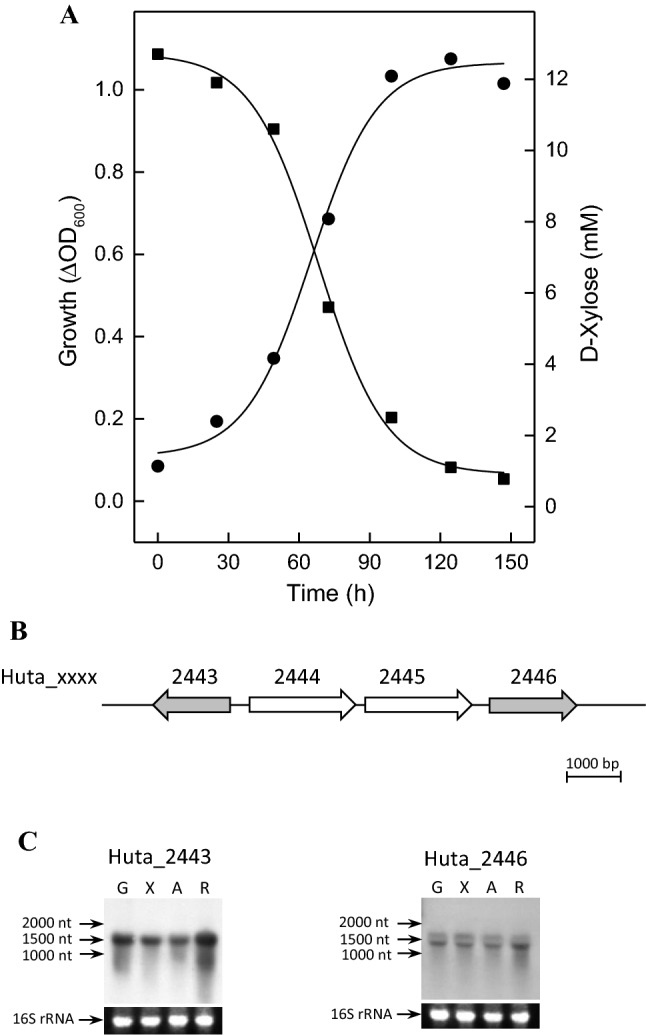
Xylose degradation in *Halorhabdus utahensis.* **a** Growth was performed at 37 °C on 15 mM D-xylose in synthetic medium (filled circle); consumption of D-xylose (filled square). **b** Genome organization of Huta_2443 and Huta_2446 encoding putative xylose isomerase and xylulokinase, respectively (arrows in grey); the genes Huta_2444 and Huta_2445 encode putative methyl-accepting chemotaxis proteins (arrows in white). **c** Northern blotting of Huta_2443 and Huta_2446 was performed with RNA from cells grown on D-glucose (G), D-xylose (X), L-arabinose (A) and D-ribose (R). 16S rRNA served as loading control

*H. utahensis* grew on d-xylose with a doubling time of 17 h up to optical densities at 600 nm of about 1.0 (Fig. 2a). Genome analysis of *H. utahensis* revealed the absence of genes encoding key enzymes of the oxidative xylose degradation to α-ketoglutarate, i.e., 2-keto-3-deoxyxylonate dehydratase and α-ketoglutarate semialdehyde dehydrogenase found in other haloarchaeal species. In addition, extracts of D-xylose-grown *H. utahensis* cells do not contain activity of α-ketoglutarate semialdehyde dehydrogenase and also of xylose dehydrogenase, the first enzyme of the oxidative degradation pathway. Instead, extracts of d-xylose-grown *H. utahensis* cells contain activities of xylose isomerase (0.049 U/mg) and xylulokinase (0.408 U/mg) and the genes Huta_2443 and Huta_2446 were identified that encode putative xylose isomerase and xylulokinase (Fig. 2b).

Transcription of Huta_2443 and Huta_2446 was followed by Northern blot analyses using RNA from cells grown on d-xylose, l-arabinose, d-ribose and d-glucose. As indicated in Fig. 2c a strong transcriptional signal at 1500 nucleotides was detected for both genes in cells grown on the three pentoses as well as on d-glucose; the transcript sizes correspond to that of Huta_2443 (1317 nucleotides) and of Huta_2446 (1548 nucleotides). The presence of transcripts in both d-glucose- and pentose-grown cells indicates that the genes are constitutively expressed. It should be noted that the genes of d-xylose degradation in bacteria are specifically regulated by d-xylose (Luo et al. 2014).

Huta_2443 and Huta_2446 were overexpressed in *Haloferax volcanii* H1209 and the recombinant enzymes were purified by affinity and size-exclusion chromatography (Supplemental Figure S1). Xylose isomerase had a molecular mass of 175 kDa; the calculated molecular mass of the subunit is 49.3 kDa, indicating a homotetrameric structure. The enzyme catalyzed the isomerization of d-xylose to xylulose showing a specific activity of 4.4 U/mg and a Km value for d-xylose of 32 mM (Supplemental Figure S2). With d-glucose as a substrate, the enzyme showed a 57-fold lower catalytic efficiency and with L-arabinose no activity (< 1%, at 100 mM) could be measured.

The homotetrameric structure of the *H. utahensis* enzyme and its significant higher catalytic efficiency of isomerization of d-xylose over d-glucose are typical features of most bacterial xylose isomerases as given in BRENDA database (https://www.brenda-enzymes.org/). It should be noted that despite their low catalytic efficiency for d-glucose, xylose isomerases of many bacteria and fungi have been characterized due to their application as “glucose isomerases” in the isomerization of d-glucose to d-fructose as part of the important biotechnological process of starch saccharification generating the sweetener high-fructose corn syrup (Bhosale et al. 1996).

Xylulokinase showed a molecular mass of 133 kDa with a calculated subunit size of 55.1 kDa indicating a homodimeric structure. In bacteria both homodimeric and homotetrameric xylulokinases have been reported (BRENDA database). The *H. utahensis* enzyme catalyzed the ATP-dependent phosphorylation of xylulose with a specific activity of 93.3 U/mg; the apparent Km values for xylulose and ATP were 0.31 mM and 5.68 mM, respectively. l-Ribulose was not phosphorylated at significant rates (< 1%, at 50 mM).

BlastP analyses with xylose isomerase and xylulokinase of *H. utahensis* each revealed hits with high sequence identity only in other *Halorhabdus* species, i.e., *H. tiamatea* (91% identity, xylose isomerase; 93%, xylulokinase) and *Halorhabdus* spec. H27 (86% identity, for each enzyme) suggesting that these haloarchaea degrade D-xylose also via the bacterial-type pathway to xylulose-5-phosphate (Table 1). No homologs of these genes were found in *Halorhabdus rudnickae*, which is in accordance with the report that this *Halorhabdus* strain is not able to grow on D-xylose (Albuquerque et al. 2016). Further, xylose isomerase and xylulokinase were not found in any other archaeal species.

**Table 1:** Enzymes and their encoding genes of bacterial-type degradation pathways of D-xylose, L-arabinose and D-ribose and of the non-oxidative pentosephosphate pathway in *Halorhabdus* species

Enzyme name	*H. utahensis*	*H. tiamatea*	*H. rudnickae*	*Halorhabdus* spec. H27
Xylose isomerase	Huta_2443	HTIA_2228	–	WP_136689821
Xylulokinase	Huta_2446	HTIA_2231	–	WP_136689820
Arabinose isomerase	Huta_1154	HTIA_1046 HTIA_p2910	–	WP_136689493
Ribulokinase	Huta_1150	HTIA_1042 HTIA_p2892	–	WP_136689520
L-Ribulose-5-phosphate-4-epimerase	Huta_1149	HTIA_1041 HTIA_p2891	–	WP_136689521
Ribokinase	–	HTIA_0439	WP_135662271	WP_136687548
Ribose-5-phosphate isomerase	Huta_0832	HTIA_0710	WP_135665987	WP_136689787
D-Ribulose-5-phosphate-3-epimerase	Huta_0833	HTIA_0711	WP_135665983	WP_136689785
Transaldolase	Huta_0859	HTIA_0717	WP_135665975	WP_136687890
Transketolase	Huta_0861 Huta_0860	HTIA_0719 HTIA_0718	WP_135665971 WP_167880040 WP_135665973	WP_169051793 WP_136687892 WP_136687891

### Sequence comparison and phylogenetic affiliation of xylose isomerase from *H. utahensis*

**Fig. 3:**
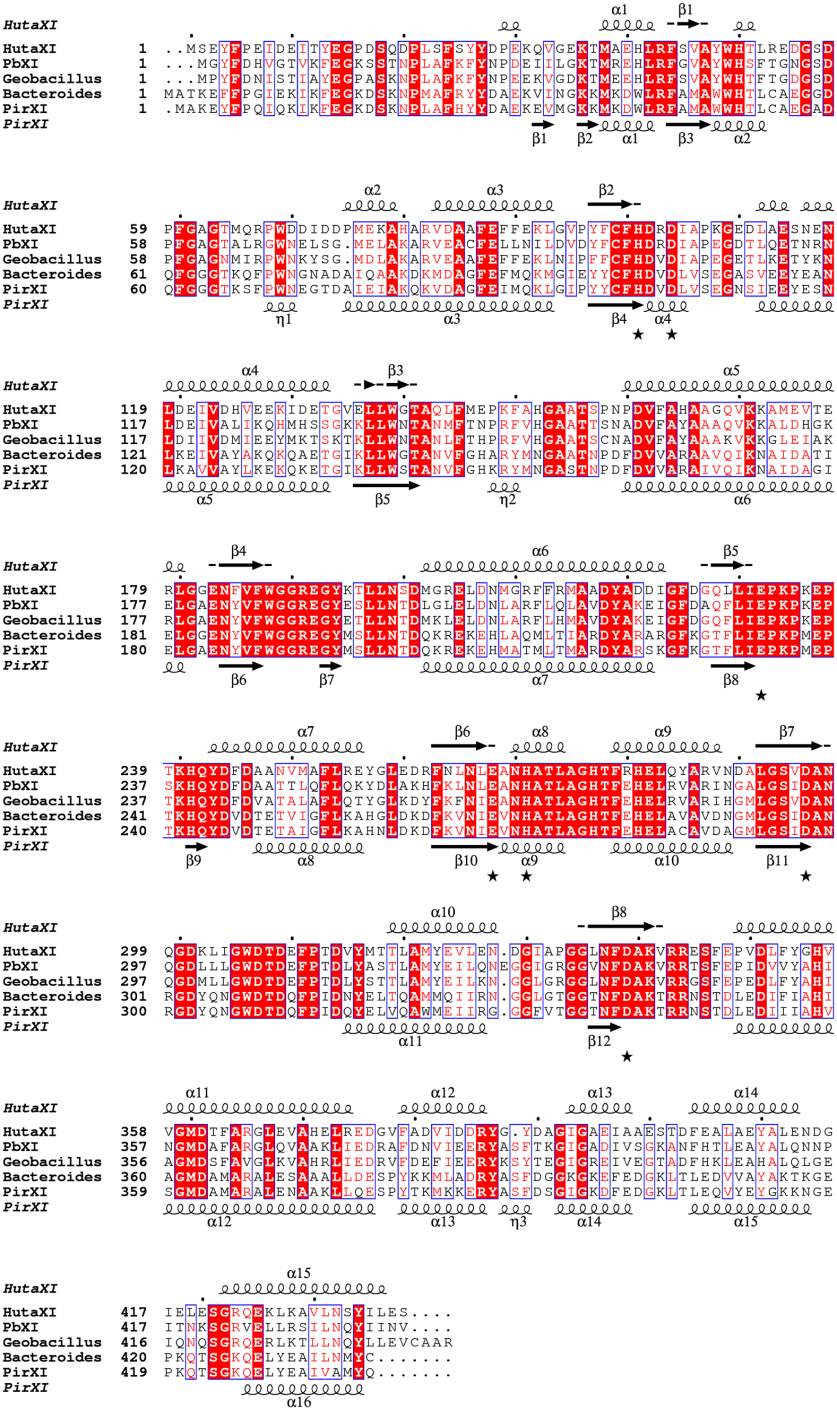
Amino acid sequence alignment of xylose isomerase from *H. utahensis* with selected xylose isomerases of class II from bacteria and fungi. Predicted secondary structure elements of the *H. utahensis* enzyme are in accordance with the structure-based secondary structure elements of *Piromyces* sp. E2 xylose isomerase (Lee et al. 2017). Conserved amino acids involved in divalent cation and substrate binding are marked by asterisks; for details (Lee et al. 2017; Son et al. 2018). PDB identifier: *Paenibacillus* sp. R4, 6INT; *Geobacillus stearothermophilus*, 1A0D; *Bacteroides thetaiotaomicron*, 4XkM; *Piromyces* sp. E2, 5NHM. UniProt entry number: *H. utahensis*, Huta_2443, C7NMH0

Xylose isomerase of *H. utahensis* showed highest sequence identity with xylose isomerases of the bacteria *Geobacillus stearothermophilus* (63%), *Thermotoga* species (58%), *Bacteroides* species (47%), and of the fungus *Piromyces* (46%); lower identities were found with the xylose isomerases of *Thermus thermophilus* and *Streptomyces* species (21–23%). Xylose isomerase of *Piromyces* and close homologs, e.g., from firmicutes and *Bacteroides* species, were characterized as class II xylose isomerases. Class II enzymes contain an extended N-terminal region and three long loop insertions, which are missing in class I enzymes (Kim et al. 2001; Son et al. 2018). These features are also present in xylose isomerase of *H. utahensis* classifying the enzyme as class II xylose isomerase. A sequence alignment of xylose isomerase of *H. utahensis* with selected class II sequences is shown in Fig. 3. The predicted secondary structure of xylose isomerase from *H. utahensis* matches well with the structure elements concluded from crystal structure of the *Piromyces* enzyme (Son et al. 2018). Further, residues involved in the catalysis are conserved in the haloarchaeal enzyme.

**Fig. 4:**
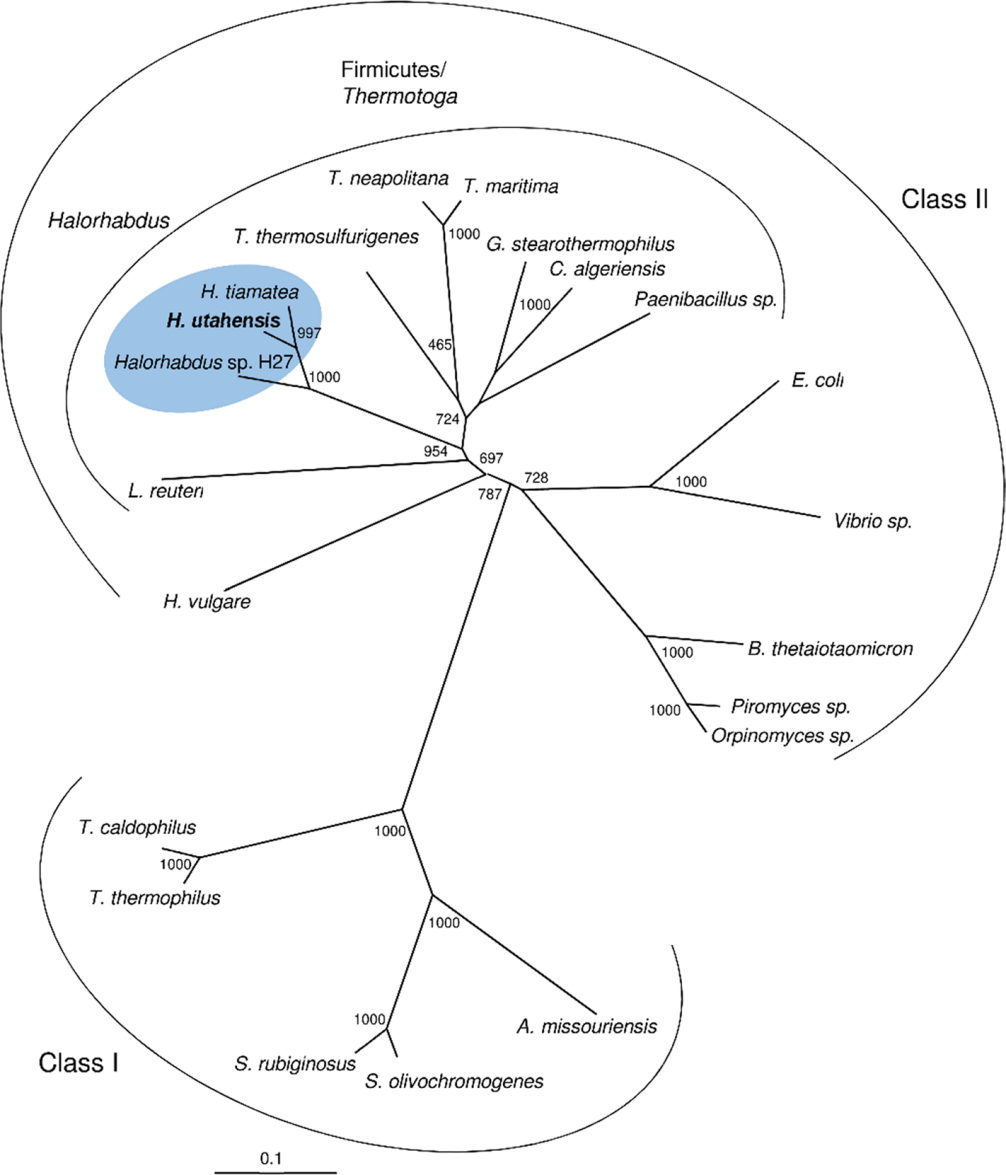
Phylogenetic relationship of xylose isomerase from *Halorhabdus* species and class I and class II xylose isomerases from bacteria and eukarya. *H. utahensis*, C7NMH0; *H. tiamatea*, S6CUC8; *Halorhabdus* sp. H27, WP_136689821; *Lactobacillus reuteri*, Q5QG16 (Staudigl et al. 2014); *Thermoanaerobacterium thermosulfurigenes*, P19148; *Thermotoga neapolitana*, P45687; *Thermotoga maritima*, Q9X1Z5 (Bandlish et al. 2002); *Geobacillus stearothermophilus*, P54273; *Caldicoprobacter algeriensis*, A0A481U6W5 (Neifar et al. 2019); *Paenibacillus* sp. R4, 6INT; *E. coli*, P00944; *Vibrio* sp., C7G532 (Umemoto et al. 2012); *Orpinomyces* sp., B7SLY1 (Madhavan et al. 2009); *Piromyces* sp., Q9P8C9 (Son et al. 2018); *Bacteroides thetaiotaomicron*, Q8A9M2 (Cho et al. 2013); *Hordeum vulgare*, Q40082; *Thermus caldophilus*, P56681; *Thermus thermophiles*, P26997; *Streptomyces olivochromogenes*, P15587; *Streptomyces rubiginosus*, P24300; *Actinoplanes missouriensis*, E6YBC0 (Wang et al. 2011)

A phylogenetic analysis was performed with xylose isomerase of *H. utahensis* and selected enzymes of class I and class II xylose isomerases (Fig. 4). The tree topology demonstrates that xylose isomerases cluster according to their affiliation to class I or II enzymes. Class I comprise xyloses isomerases from *Streptomyces* and *Thermus* species and class II xylose isomerases from firmicutes, *Thermotoga* species, proteobacteria, bacteroides, *Hordeum vulgare* and fungi. Xylose isomerase from *H. utahensis* belongs to the class II cluster in accordance with the sequence alignment (Fig. 3). Within the class II cluster, the haloarchaeal enzymes form a sub-cluster together with firmicutes and *Thermotoga* species. Together, the finding that the xylose isomerase of *H. utahensis* is closely related to firmicutes/*Thermotoga* xylose isomerases, and the absence of xylose isomerases in any other archaeal species suggests that xylose isomerase in *Halorhabdus* species has been acquired from firmicutes via lateral gene transfer.

### L-Arabinose degradation to xylulose-5-phosphate in *H. utahensis*

**Fig. 5:**
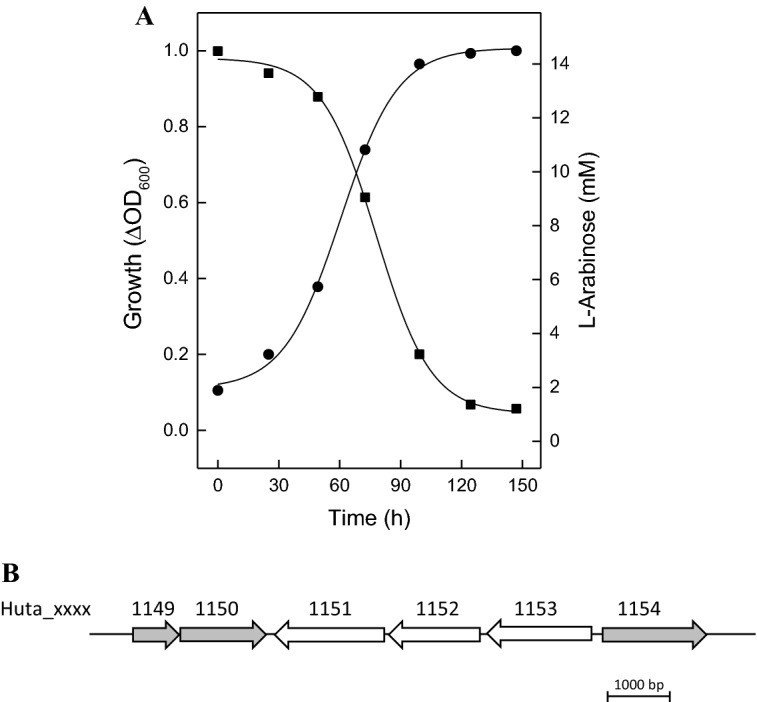
L-Arabinose degradation in *Halorhabdus utahensis*. **a** Growth of *H. utahensis* on L-arabinose. Growth was performed at 37 °C on 15 mM L-arabinose in synthetic medium (filled circle); consumption of L-arabinose (filled square). **b** Genome organization of Huta_1154, Huta_1150 and Huta_1149 encoding putative arabinose isomerase, ribulokinase and L-ribulose-5-phosphate-4-epimerase (grey arrows)

*H. utahensis* grew on L-arabinose with a doubling time of 18 h up to an optical density at 600 nm of 1.0 (Fig. 5a). In cell extracts of L-arabinose-grown cells, activities of arabinose dehydrogenase and α-ketoglutarate dehydrogenase could not be detected excluding an oxidative pathway of l-arabinose degradation in *H. utahensis.* Instead, the organism contains genes that encode putative arabinose isomerase (Huta_1154), ribulokinase (Huta_1150) and l-ribulose-5-phosphate-4-epimerase (Huta_1149), i.e., enzymes of the classical non-oxidative l-arabinose degradation pathway found in most bacteria. (Fig. 5b).

Transcription of Huta_1154 and Huta_1150 was followed by Northern blot analyses using RNA from cells grown on d-xylose, l-arabinose, d-ribose and d-glucose. A signal at about 1600 nucleotides was detected in all RNAs using a Huta_1154 specific probe that matches well to the gene length of 1488 nucleotides (not shown). With a probe against Huta_1150, two signals were detected in pentose- and glucose-grown cells, at about 2200 and 1300 nucleotides, indicating cotranscription of Huta_1150 (1539 nucleotides) and Huta_1149 (648 nucleotides), as well as single transcription of Huta_1150 (Supplemental Figure S3). The data indicate that the three genes are constitutively expressed during growth on d-glucose and the three pentoses. In contrast, l-arabinose degradation in bacteria has been reported to be specifically regulated by l-arabinose (Luo et al. 2014).

Huta_1154 was expressed and the recombinant arabinose isomerase was purified (Supplemental Figure S1) as 322 kDa enzyme, the calculated molecular mass of the subunits is 55.4 kDa indicating a homohexameric structure. The specific activity was 129.5 U/mg; the apparent Km value was 254.6 mM for l-arabinose. No activity was measured with d-galactose, d-arabinose, d-ribose and d-xylose (tested at 10 and 100 mM).

The oligomerization of arabinose isomerase of *H. utahensis* as homohexamer has been reported for few bacterial and eukaryal enzymes, e.g., from *E. coli* (Patrick and Lee 1969) and from *Arthrobacter* sp. (Wanarska and Kur 2012); however, the majority of arabinose isomerases, e.g., from *Bacillus* species, were characterized as homotetrameric enzymes (Wanarska and Kur 2012).

The arabinose isomerase of *H. utahensis* is highly specific for the isomerization of L-arabinose showing no activity with d-galactose. Thus, the enzyme differs from most characterized arabinose isomerases from eukarya and bacteria which catalyze—in addition to l-arabinose—the isomerization of d-galactose to d-tagatose. Since d-tagatose is used as a low-calorie sugar substitute in food industry, arabinose isomerases are well-studied enzymes in biotechnology (Kim 2004). However, few bacterial arabinose isomerases have been described, e.g., from *Bacillus* species, that also have a high specificity for l-arabinose over d-galactose as the *Halorhabdus* enzyme (Li et al. 2011; Prabhu et al. 2008).

Homologs of arabinose isomerase were found in other *Halorhabdus* species, e.g., *Halorhabdus tiamatea* and *Halorhabdus* spec. H27, rather than in *Halorhabdus rudnickae* (Table 1). Further, the haloarchaeal arabinose isomerase shows high sequence identity (53%) to homologs of bacteria, e.g., *E. coli* and *Bacillus subtilis*.

The recombinant ribulokinase encoded by Huta_1150 was purified (Supplemental Figure S1) as enzyme of 348 kDa; the calculated molecular mass of subunit is 56.1 kDa indicating a homohexameric structure. The enzyme catalyzed the ATP-dependent phosphorylation of L-ribulose with a specific activity of 60.5 U/mg; the apparent Km values of L-ribulose and ATP were 0.55 mM and 4.2 mM, respectively; D-xylulose was not used as substrate. Ribulokinase shows high sequence identity to homologs in *H. tiamatea* (95% identity) and *Halorhabdus* spec. H27 (83%) rather than in *H. rudnickae* (Table 1).

Huta_1149 encoding L-ribulose-5-phosphate-4-epimerase forms a cotranscript with Huta_1150 (Fig. 5, Supplemental Figure S3). In bacteria, L-ribulose-5-phosphate-4-epimerases catalyze the conversion of ribulose-5-phosphate to D-xylulose-5-phosphate. Putative homologs of this enzyme were only found in *Halorhabdus* species *H. tiamatea* and *Halorhabdus* sp. H27 (Table 1). Together, we propose that these *Halorhabdus* species degrade L-arabinose via the classical bacterial-type pathway.

###  d-Ribose degradation to xylulose-5-phosphate in Halorhabdus species

**Fig. 6:**
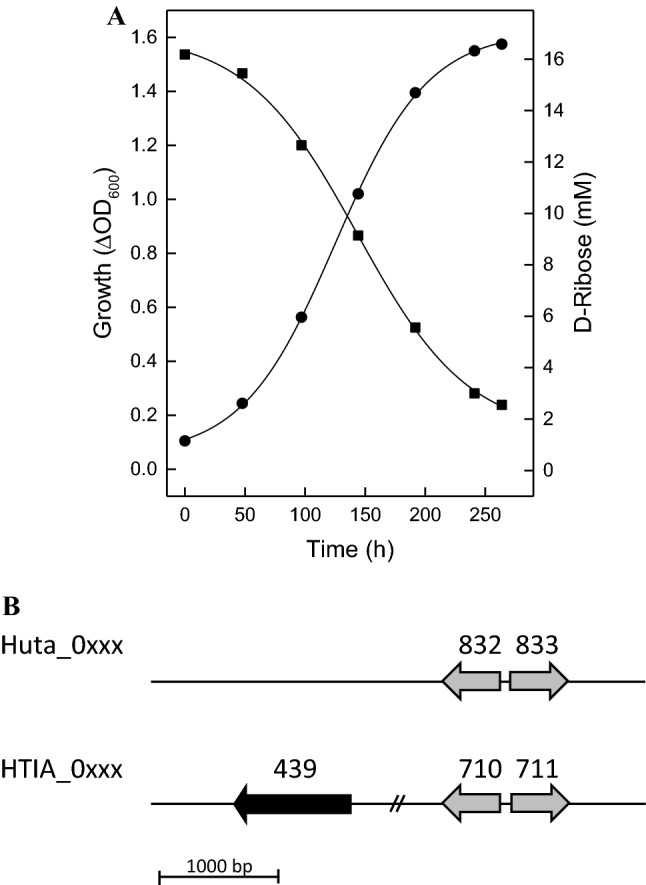
D-Ribose degradation in *Halorhabdus* species *H. utahensis* and *H. tiamatea*. **a** Growth of *H. utahensis* was performed at 37 °C on 15 mM D-ribose in synthetic medium (filled circle); consumption of D-ribose (filled square). **b** Genomic view of genes involved in D-ribose degradation in *H. utahensis* and *H. tiamatea*. A homologous gene of HTIA_0439 (black arrow) encoding ribokinase of *H. tiamatea* is absent in *H. utahensis*. HTIA_0710 and Huta_0832 encode ribose-5-phosphate isomerase and HTIA_0711 and Huta_0833 encode D-ribulose-5-phosphate-3-epimerase (grey arrows)

*H. utahensis* grew on d-ribose with a doubling time of 29 h up to optical densities at 600 nm of about 1.6 (Fig. 6). A gene encoding ribonate dehydratase, the key enzyme of oxidative d-ribose degradation in *Haloarcula* species, is not present in genomes of *Halorhabdus* species. Instead, genes were identified that encode putative enzymes of the classical non-oxidative d-ribose degradation pathway operative in most bacteria. In the genome of *H. utahensis* two clustered genes, Huta_0832 and Huta_0833, encoding putative ribose-5-phosphate isomerase and d-ribulose-5-phosphate-3-epimerase are annotated; a gene homologous to ribokinase from *E. coli* could not be identified. In contrast to *H. utahensis*, we found that the *Halorhabdus* species *H.tiamatea, Halorhabdus* sp. H27 and *H. rudnickae* each contain a bacterial type ribokinase gene in addition to the genes encoding ribose-5-phosphate isomerase and D-ribulose-5-phosphate-3-epimerase (Fig. 6; Table 1).

**Fig. 7:**
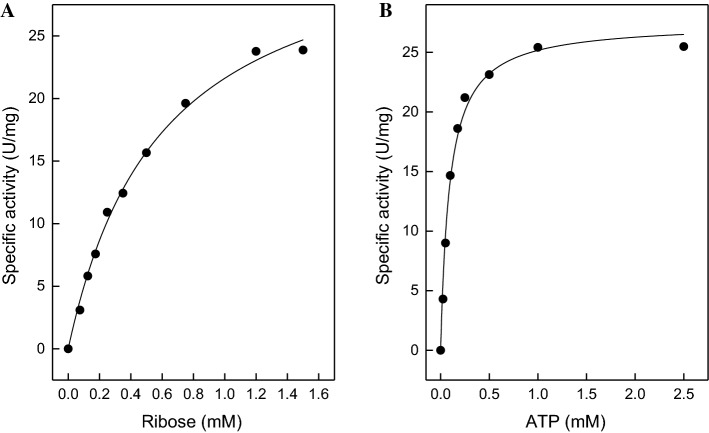
Rate dependence of ribokinase of *H. tiamatea* on the concentrations of ribose and ATP

HTIA_0439 encoding putative ribokinase in *H. tiamatea* was overexpressed and the recombinant protein was purified. Ribokinase was characterized as dimeric protein of 70 kDa composed of 31.3 kDa subunits (Supplemental Figure S4). The enzyme catalyzed the ATP-dependent phosphorylation of d-ribose with a specific activity of 34.5 U/mg and apparent Km values of d-ribose and ATP of 0.6 mM and 0.1 mM, respectively (Fig. 7).

Despite the absence of a bacterial ribokinase gene in *H. utahensis*, d-ribose-grown cells showed low ribokinase activity (~ 1 mU/mg). To identify the encoding gene we purified ribokinase activity up to 1300-fold (Supplemental Figure S5). The purified ribokinase showed a single subunit on SDS-PAGE of 75 kDa and by MALDI-TOF analysis Huta_1150 was identified as the encoding gene; this gene encodes ribulokinase involved in L-arabinose degradation (see above). We reanalyzed the kinetic properties of ribulokinase and found that it catalyzed the phosphorylation of d-ribose with a specific activity of 1.3 U/mg. We propose that in *H. utahensis* the phosphorylation of d-ribose is catalyzed by ribulokinase that functionally replaces the missing ribokinase as first enzyme of the d-ribose degradation pathway.

### Sequence comparison and phylogenetic affiliation of ribokinase from *H. tiamatea*

**Fig. 8:**
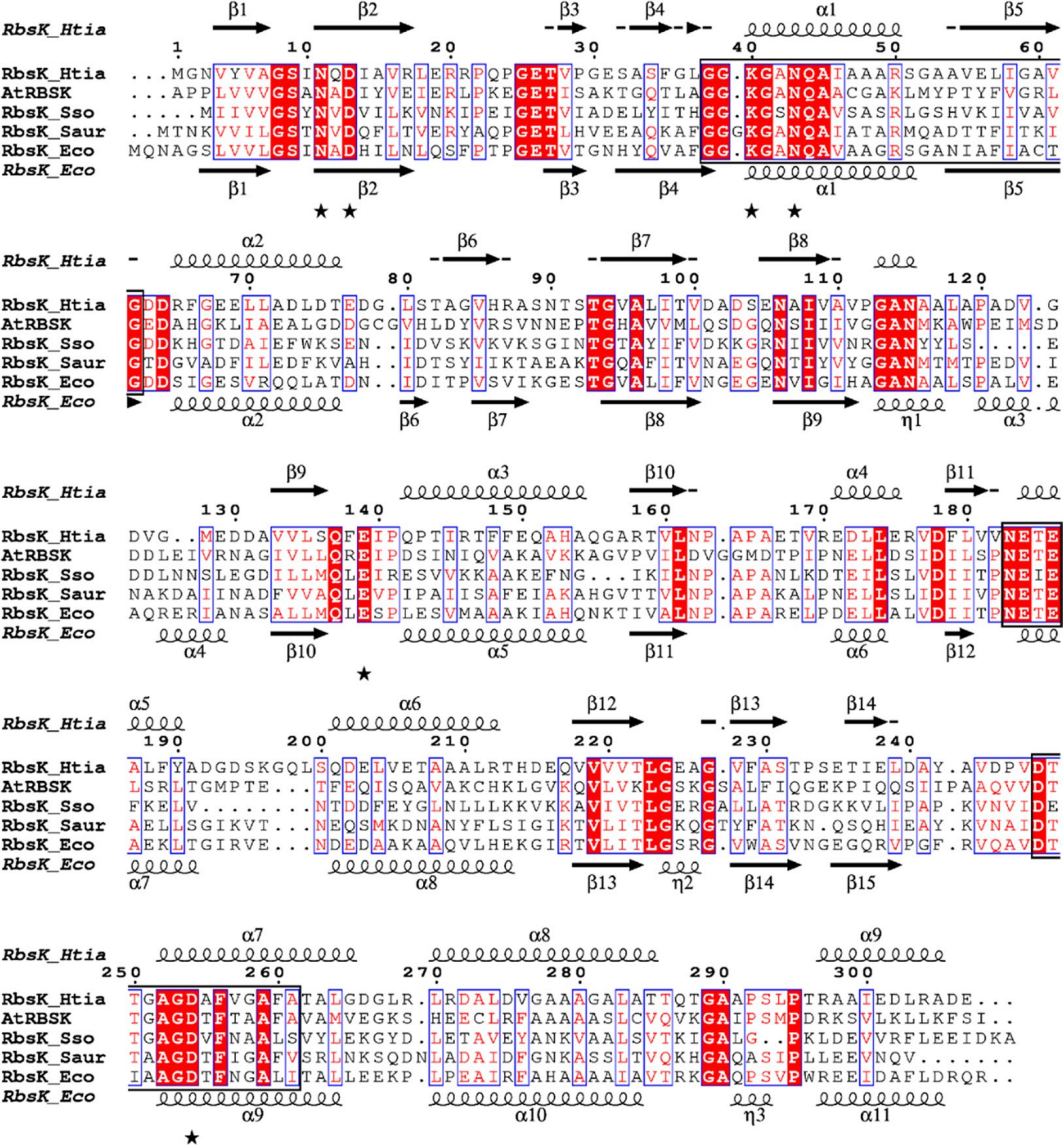
Amino acid sequence alignment of ribokinase from *H. tiamatea* with selected characterized and putative ribokinases from bacteria, archaea and eukarya. Highly conserved residues are shown in red and boxed in blue; strictly conserved residues are shown with a red background. The two signatures (PDOC00504) of pfkB family of carbohydrate kinases and the typical ribokinase family NXXE motif (Park and Gupta 2008) are boxed. Predicted secondary structure elements of *H. tiamatea* ribokinase match well with the structure-based secondary structure elements of *E. coli* ribokinase (Andersson and Mowbray 2002). Conserved amino acids involved in ribose binding are marked by asterisks according to the structures from *E. coli* and *Arabidopsis thaliana* (Andersson and Mowbray 2002; Kang et al. 2019). PDB identifier: *E. coli*, 1GQT; *A. thaliana*, the first 69 residues are not shown, 6ILT; *S. aureus*, 3RY7. UniProt entry number: *H. tiamatea*, HTIA_0439, C7NMH0; *Sulfolobus solfataricus*, Q981E2

The ribokinase from *H. tiamatea* shows significant sequence identity with putative ribokinases from the *Halorhabdus* species *H. rudnickae* (59%) and *Halorhabdus* sp. H27 (78%) and at lower identity from other archaea, e.g., *Sulfolobus solfataricus* (29%) and *Ferroplasma acidiphilum* (32%). *H. tiamatea* ribokinase also shows significant sequence identity with characterized ribokinases from bacteria and eukarya, including *Escherichia coli* (37%), *Staphylococcus aureus* (34%), human (30%) and *Arabidopsis thaliana* (28%). A sequence alignment of ribokinase of *H. tiamatea* and selected ribokinases characterized from bacteria and eukarya, and the putative archaeal ribokinase from *S. solfataricus* is shown in Fig. 8. The predicted secondary structure of ribokinase from *H. tiamatea* matches well with the structure elements of *E. coli* ribokinase (Andersson and Mowbray 2002). Further, residues that are involved in binding of d-ribose based on crystal structures of *E. coli* and *A. thaliana* are conserved in the haloarchaeal enzyme (Andersson and Mowbray 2002; Kang et al. 2019). Ribokinases belong to the PfkB family of carbohydrate kinases (Park and Gupta 2008) and sequences of this family contain the two typical sequence pattern of PfkB family (Fig. 8). Further, the alignment demonstrates the similarity of characterized ribokinases and the putative ribokinase from *S. solfataricus*. The residues for d-ribose binding are conserved in *Sulfolobus* enzyme suggesting that it also shows ribokinase activity; yet, its catalytic activity and functional involvement in d-ribose phosphorylation as part of a d-ribose degradation pathway in *Sulfolobus* have to be demonstrated.

**Fig. 9:**
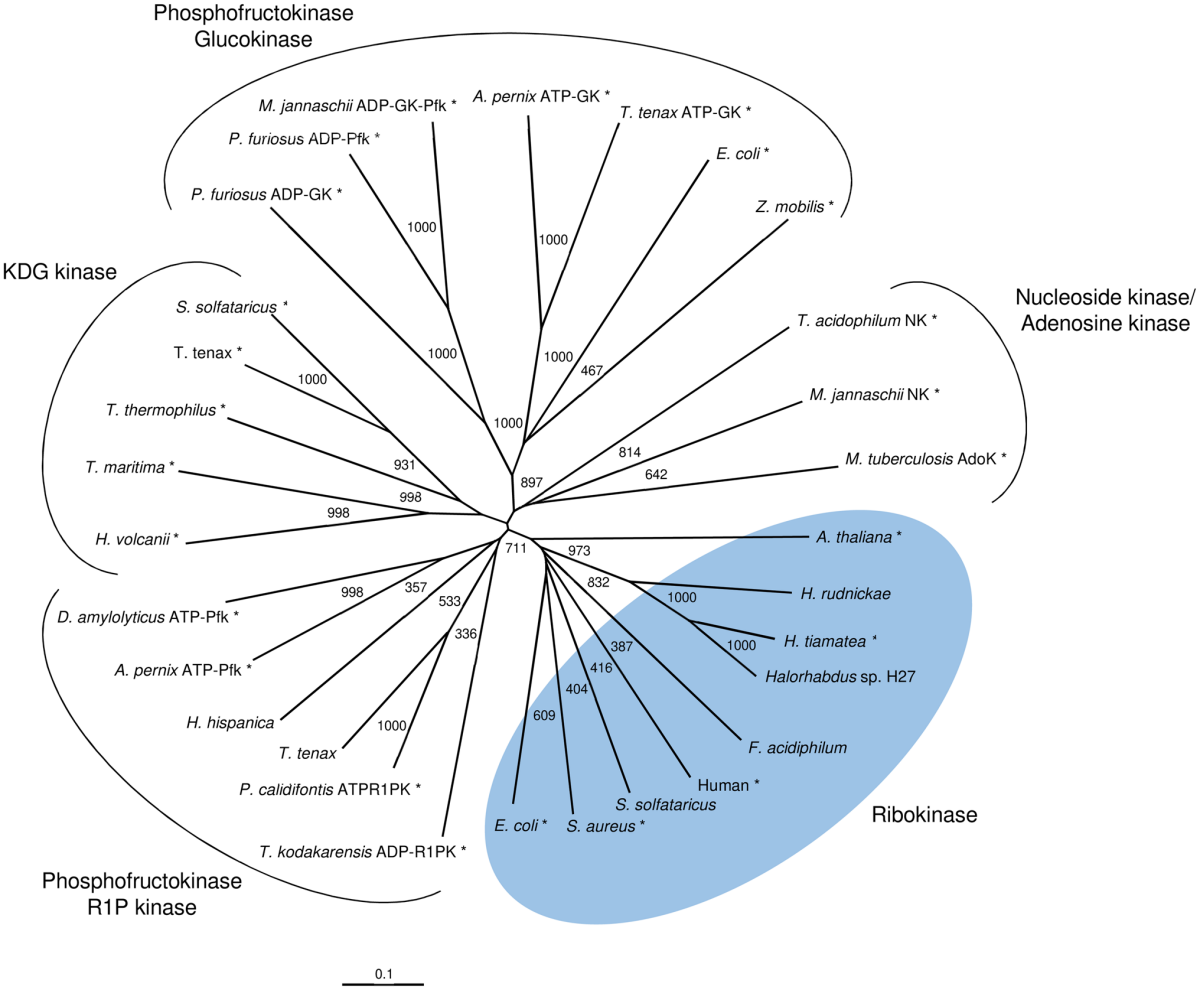
Phylogenetic relationship of ribokinase from *H. tiamatea* with ribokinases from bacteria, eukarya and archaea and related members of the ribokinase superfamily. Ribokinase: *Halorhabdus tiamatea*, HTIA_0439; *Halorhabdus rudnickae*, WP_135662271; *Halorhabdus* sp. H27, WP_136687548; *Arabidopsis thaliana*, A1A6H3; *Ferroplasma acidiphilum*, A0A1V0N6I9; *Sulfolobus solfataricus*, SSO0004; Human, Q9H477; *E. coli*, P0A9J6; *Staphylococcus aureus*, A0A0H2WZY4; Nucleoside/adenosine kinase: *Thermoplasma acidophilum*, Ta0880; *Methanocaldococcus jannaschii*, MJ0406; *Mycobacterium tuberculosis*, P9WID5; Phosphofructokinase/ribose-1-phosphate kinase: *Desulfurococcus amylolyticus*, WP_042667458 (Hansen and Schönheit 2000); *Aeropyrum pernix*, Ape0012 (Hansen and Schönheit 2001); *Thermoproteus tenax*, TTX_1882; *Pyrobaculum calidifontis*, Pcal 0041 (Aziz et al. 2018); *Haloterrigena hispanica*, A0A1G6RMF8; *Thermococcus kodakarensis*, TK2029 (Aono et al. 2015); Glucokinase/phosphofructokinase: *T. tenax*, TTX0060 (Dörr et al. 2003); *A. pernix*, Ape2091; *Zymomonas mobilis*, Q03417 (King et al. 1996); *E. coli*, P23917; *M. jannaschii*, MJ1604; *Pyrococcus furiosus*, PF1784 (ADP-Pfk); *P. furiosus*, PF0312 (ADP-GK); 2-Keto-3-deoxygluconate kinase: *Haloferax volcanii*, HVO_0549; *Thermotoga maritima*, Q9WXS2 (Mathews et al. 2008); *Thermus thermophilus*, Q53W83; *T. tenax*, TTX_1157 (Ahmed et al. 2005); *S. solfataricus*, SSO3195

Ribokinase from *H. tiamatea* and archaeal homologs from other *Halorhabdus* species and from *S. solfataricus* and *F. acidiphilum* are members of the ribokinase superfamily (PfkB family of carbohydrate kinases). A phylogenetic relationship between ribokinases and four selected families of the ribokinase superfamily is shown in Fig. 9. The tree topology shows that ribokinases and the members of other families each form a distinct cluster according to their various kinase functions as follows: (1) the ribokinase cluster includes characterized and putative enzymes from eukarya, bacteria and archaea, whereby the *Halorhabdus tiamatea* enzyme represents the first characterized ribokinase in the archaeal domain. (2) The phosphofructokinase/ribose-1-phosphate kinase cluster includes archaeal ADP- and ATP-dependent ribose-1-phosphate kinases from *T. kodakarensis* and *P. calidifontis* and archaeal ATP-dependent phosphofructokinases from *A. pernix* and *D. amylolyticus*. Further, putative proteins from the haloarchaeon *H. hispanica*, and from *T. tenax* belong to this cluster. (3) The glucokinase/phosphofructokinase cluster includes ADP-dependent glucokinases and phosphofructokinases from hyperthermophilic euryarchaeota, *P. furiosus* and *M. jannaschii*, fructokinases from the bacteria *E. coli* and *Z. mobilis* and ATP-dependent glucokinases from the crenarchaeota *A. pernix* and *T. tenax.* (4) The nucleoside kinase/adenosine kinase cluster includes adenosine kinase from the bacterium *M. tuberculosis*, a nucleoside kinase from the archaeon *M. jannaschii* and a putative homolog from *T. acidophilum*. (5) The KDG kinase cluster includes KDG kinases from the bacteria *T. maritima* and *T. thermophilus*, from the archaea *S. solfataricus* and *T. tenax* and from the haloarchaeon *H. volcanii*.

A similar topology of members of the ribokinase superfamily from archaea, bacteria and eukarya has been reported previously (Aziz et al. 2018; Hansen et al. 2007). With the characterization of the ribokinase of *H. tiamatea*, we expand the ribokinase cluster by the first characterized archaeal member.

Huta_0832 of *H. utahensis* encodes a putative ribose-5-phosphate isomerase of the RpiA family. Transcript analysis of Huta_0832 (687 nucleotides) was performed by Northern blotting with RNA from cells grown on d-xylose, d-ribose and l-arabinose as compared to d-glucose. A specific transcript signal at 800 nucleotides could be detected in d-glucose- and pentose-grown cells indicating that Huta_0832 was constitutively expressed on these sugars (Supplemental Figure S6). Huta_0832 was overexpressed and the purified enzyme was characterized as 98.5 kDa homotetrameric protein composed of 23.9 kDa subunits (Supplemental Figure S1). The enzyme showed a specific activity of 291.6 U/mg and a Km value of D-ribose-5-phosphate of 7.64 mM; d-glucose and d-xylose were not used. Huta_0833 encodes a putative d-ribulose-5-phosphate-3-epimerase that in bacteria catalyzes the conversion of d-ribulose-5-phosphate to d-xylulose-5-phosphate. Homologs of ribose-5-phosphate isomerase and d-ribulose-5-phosphate-3-epimerase from *H. utahensis* were also found in *Halorhabdus* species *H. tiamatea*, *H. rudnickae* and *Halorhabdus* strain H27 (Table 1).

In summary, we conclude that *Halorhabdus utahensis* degrades the pentoses via the bacterial-type pathways yielding d-xylulose-5-phosphate. A comparative analysis of genomes of four *Halorhabdus* species, *H. utahensis*, *H.tiamatea*, *H. rudnickae* and *Halorhabdus* strain H27 revealed the presence of all genes of the bacterial pentose degradation pathways with few variations: *H. utahensis* does not contain ribokinase but rather utilizes l-ribulokinase for d-ribose phosphorylation, and the genes of d-xylose and l-arabinose degradation are absent in *H. rudnickae* (Table 1). Further degradation of xylulose-5-phosphate in the *Halorhabdus* species likely involves the enzymes of the non-oxidative branch of the pentose phosphate pathway (NOPP), i.e., d-ribulose-5-phosphate-3-epimerase, ribose-5-phosphate isomerase, transketolase and transaldolase, to generate the glycolytic intermediates fructose-6-phosphate and glyceraldehyde-3-phosphate (Table 1). These intermediates are further oxidized to CO_2_ to generate ATP involving enzymes of the Embden–Meyerhof pathway (Anderson et al. 2011), citric acid cycle and respiratory chain. Beside its role in pentose catabolism the NOPP pathway is also involved in the formation of sugar phosphates in anabolism.

### Bacterial-type versus archaeal-type of pentose degradation in haloarchaea

In this study, we showed that *Halorhabdus* species degrade pentoses via bacterial-type pathways yielding xylulose-5-phosphate. The exclusive presence of these pathways in *Halorhabdus* species and the absence in other archaea indicate that the pentose degradation pathways have been acquired by *Halorhabdus* from bacteria via horizontal gene transfer. Thus, *Halorhabdus* species utilizing the bacterial-type pentose degradation pathway generating xylulose-5-phosphate differ from other haloarchaea, i.e., *Haloferax* and *Haloarcula* species which degrade pentoses via the archaeal oxidative pathway yielding α-ketoglutarate, an intermediate of the citric acid cycle. The reason why *Halorhabdus* species do not use the oxidative pathway generating α-ketoglutarate might be explained by an incomplete gluconeogenetic pathway reported for *Halorhabdus* species. *Halorhabdus* species lack the enzymes that convert α-ketoglutarate via malate to the central intermediate phosphoenolpyruvate; these include malic enzyme, phosphoenolpyruvate synthetase/pyruvate phosphate dikinase or phosphoenolpyruvate carboxykinase. Thus, utilization of α-ketoglutarate in the anabolism is prevented. In contrast, *Haloferax* and *Haloarcula* species that use the oxidative pathway contain all enzymes of gluconeogenesis, which—together with malic enzyme and phosphoenolpyruvate synthetase—catalyze the conversion of α-ketoglutarate to phosphoenolpyruvate. Thus, we conclude that the utilization of different types of pentose catabolic pathways in haloarchaea is due to the different pathways in the anabolism.

## Electronic supplementary material

Below is the link to the electronic supplementary material.

Supplementary file1 (docx 3765 kb)
