# Correction to: Pentose degradation in archaea: *Halorhabdus* species degrade D-xylose, L-arabinose and D-ribose via bacterial-type pathways

**DOI:** 10.1007/s00792-021-01248-7

**Published:** 2021-11-01

**Authors:** Jan-Moritz Sutter, Ulrike Johnsen, Andreas Reinhardt, Peter Schönheit

**Affiliations:** Institut für Allgemeine Mikrobiologie, Christian-Albrechts-Universität Kiel, Am Botanischen Garten 1-9, 24118 Kiel, Germany

## Correction to: Extremophiles (2021) 24:759–772 10.1007/s00792-020-01192-y

The article “Pentose degradation in archaea: Halorhabdus species degrade D-xylose, L-arabinose and D-ribose via bacterial-type pathways”, written by “Jan‑Moritz Sutter, Ulrike Johnsen, Andreas Reinhardt, Peter Schönheit”, was originally published Online First without Open Access. After publication in volume 24, issue 5, page 759–772 the author decided to opt for Open Choice and to make the article an Open Access publication. Therefore, the copyright of the article has been changed to © The Author(s) 2021 and this article is licensed under a Creative Commons Attribution 4.0 International License, which permits use, sharing, adaptation, distribution and reproduction in any medium or format, as long as you give appropriate credit to the original author(s) and the source, provide a link to the Creative Commons licence, and indicate if changes were made. The images or other third party material in this article are included in the article’s Creative Commons licence, unless indicated otherwise in a credit line to the material. If material is not included in the article’s Creative Commons licence and your intended use is not permitted by statutory regulation or exceeds the permitted use, you will need to obtain permission directly 30 from the copyright holder. To view a copy of this licence, 31 visit http://creativecommons.org/licenses/by/4.0/.

The original article has been corrected.

