# Supplementary material for: Pentose degradation in archaea: Halorhabdus species degrade D-xylose, L-arabinose and D-ribose via bacterial-type pathways
Source: Extremophiles. 2020 Aug 5;24(5):759–72. doi: 10.1007/s00792-020-01192-y (PMC8551123; doi:10.1007/s00792-020-01192-y)
Supplement: Supplementary file 1 — Supplementary file1 (docx 3765 kb) [file 792_2020_1192_MOESM1_ESM.docx]

**Supplementary information**

**Pentose degradation in archaea: *Halorhabdus* species degrade D-xylose, L-arabinose and D-ribose via bacterial type pathways**

Jan-Moritz Sutter^1^, Ulrike Johnsen^1^, Andreas Reinhardt^1^ and Peter Schönheit^1*^

^1^Institut für Allgemeine Mikrobiologie, Christian-Albrechts-Universität Kiel

Am Botanischen Garten 1-9; D-24118 Kiel, Germany

**Table of contents**

**Figure S1** Purified recombinant enzymes from *Halorhabdus utahensis*.

**Figure S2** Rate dependence of xylose isomerase from *H. utahensis* on the D-xylose concentration.

**Figure S3** Transcriptional analyses of genes involved in L-arabinose degradation from *H. utahensis*.

**Figure S4** Purified ribokinase from *Halorhabdus tiamatea*.

**Figure S5** Purification of L-ribulose kinase showing ribokinase activity from *H. utahensis* cells grown on D-ribose.

**Figure S6** Transcriptional analysis of Huta_0832 from *H. utahensis*.

**Table S1** Plasmids used in this study.

**Supplemental Figure S1** Purified recombinant enzymes from *Halorhabdus utahensis*. Coomassie blue stained SDS-PAGE. (A) xylose isomerase, (B) xylulokinase, (C) arabinose isomerase, (D) ribulokinase, (E) ribose-5-phosphate isomerase. Lane: 1, molecular mass standard; 2, recombinant protein.

**A B**


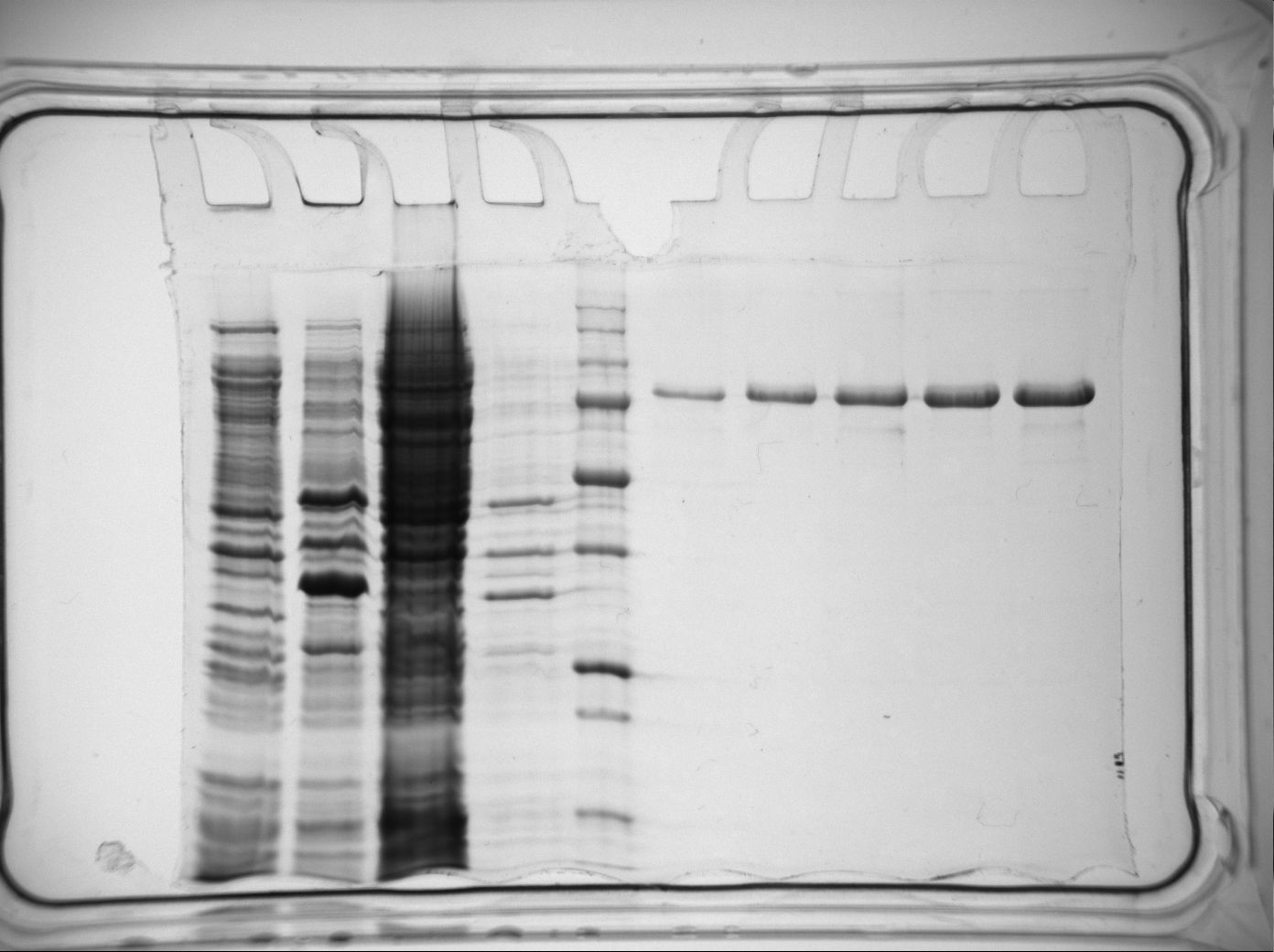

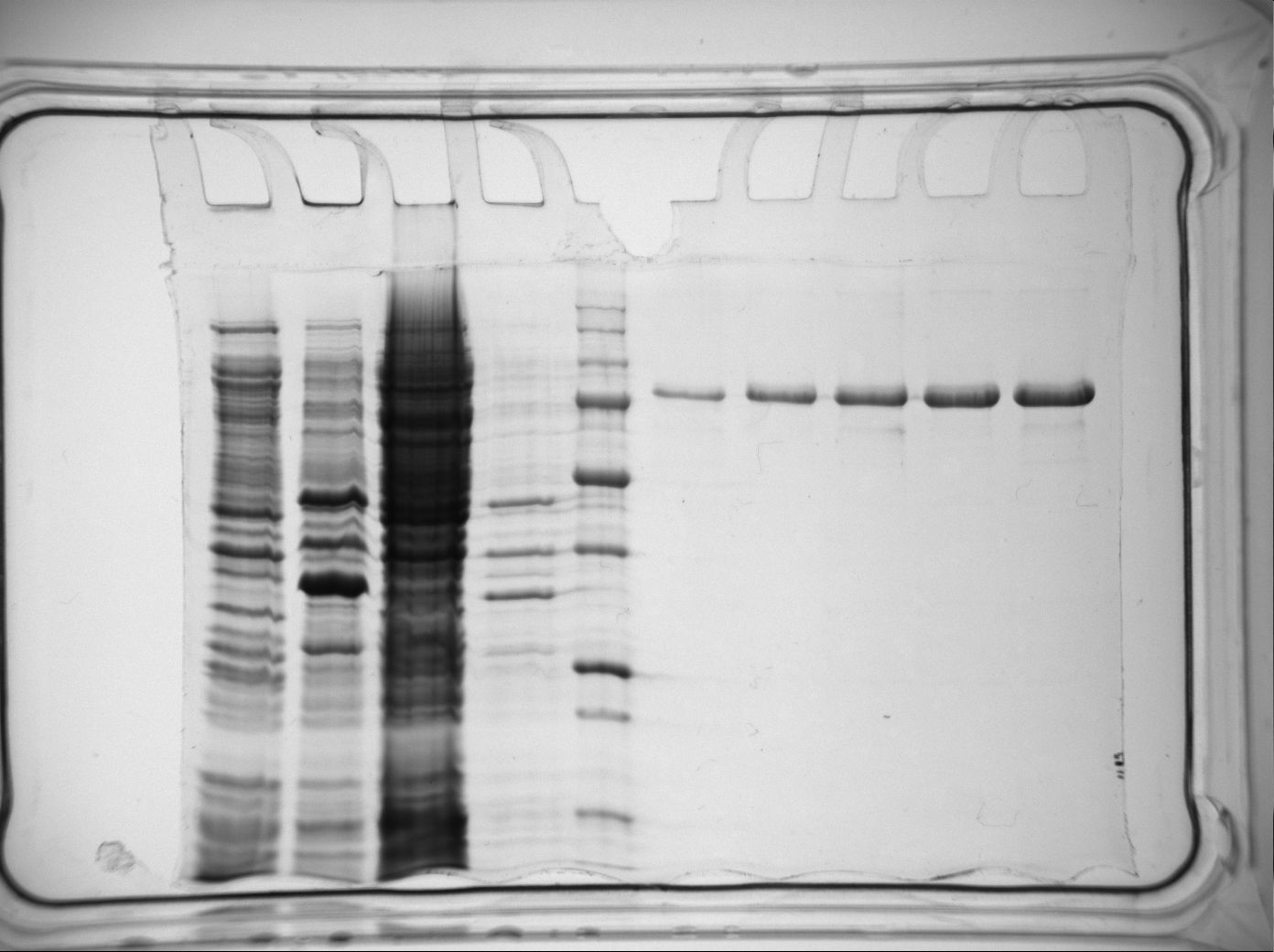


76 kDa

75

50

37

kDa

1

2


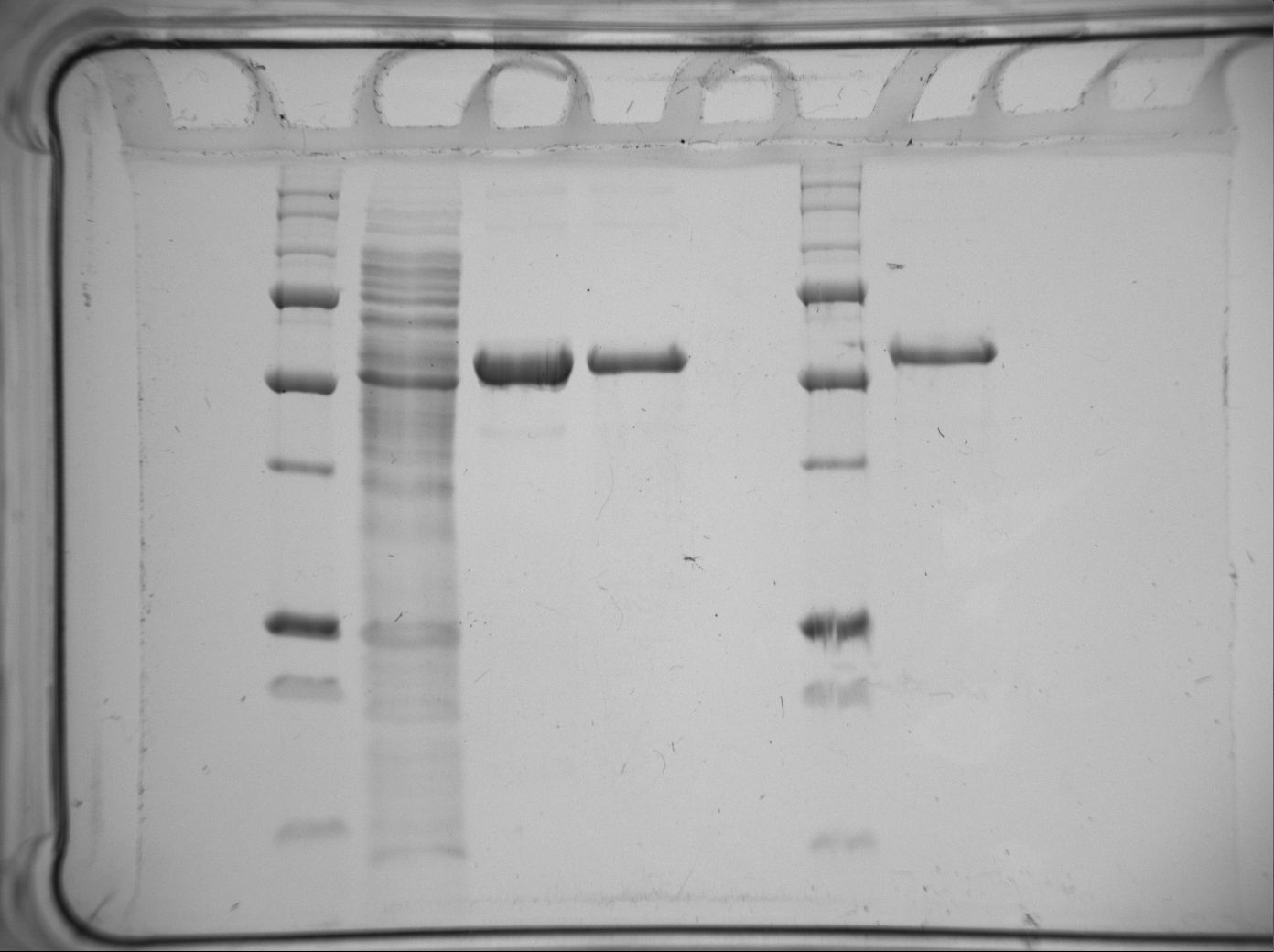


56 kDa

75

50

37

kDa

1

2

**C D**


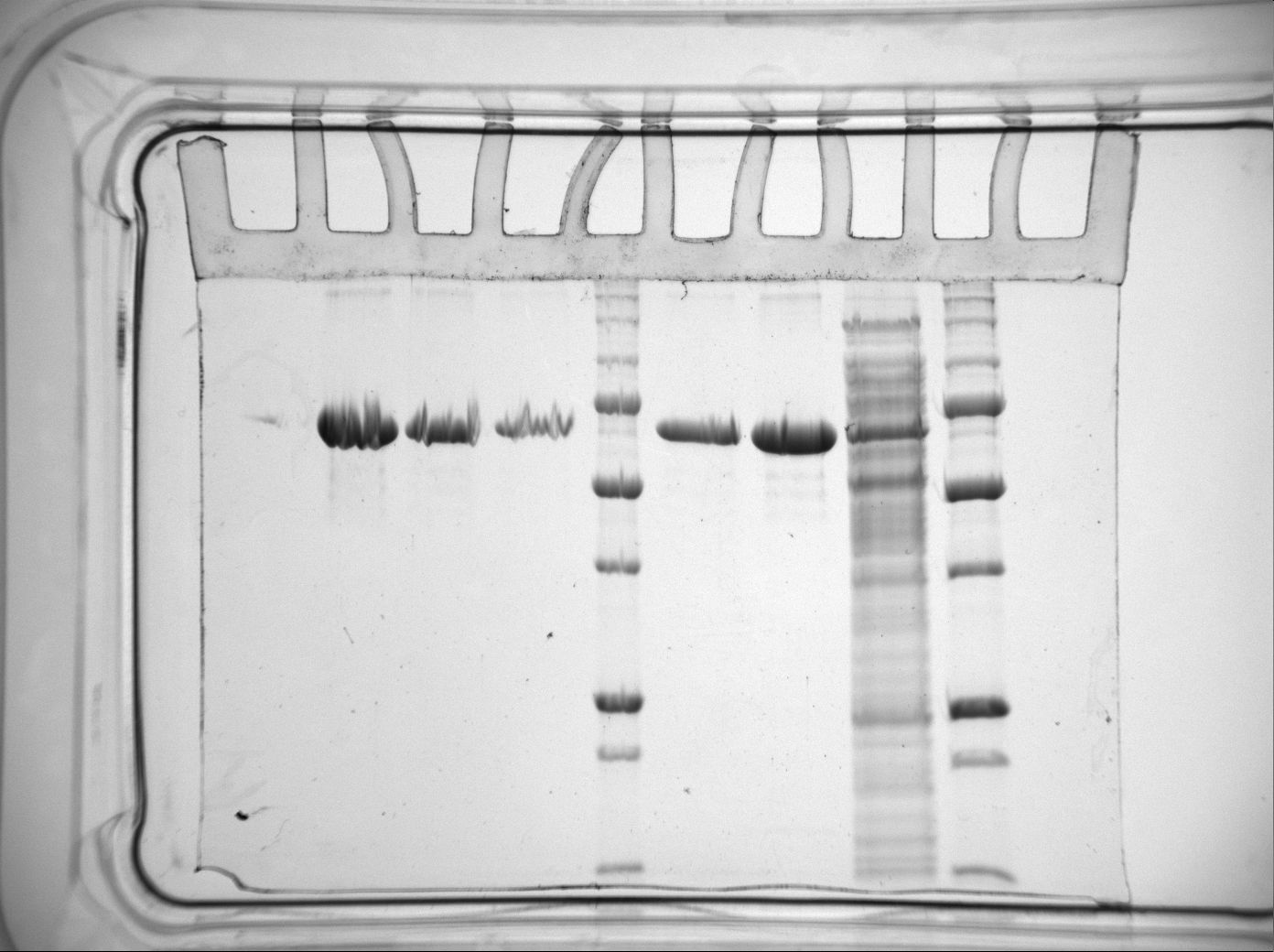


68 kDa

75

50

37

kDa

1

2


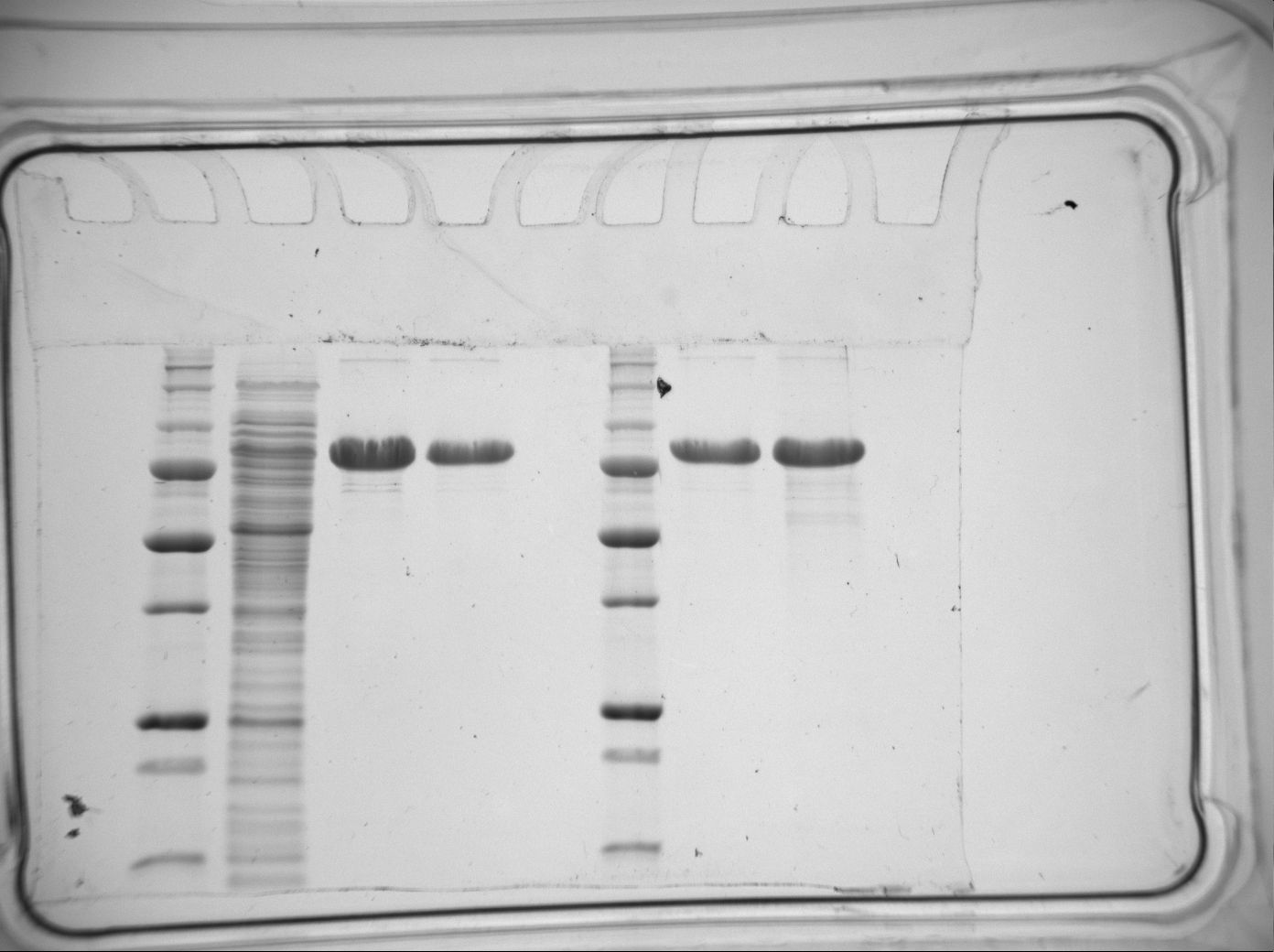


84 kDa

75

50

37

kDa

1

2

**E**


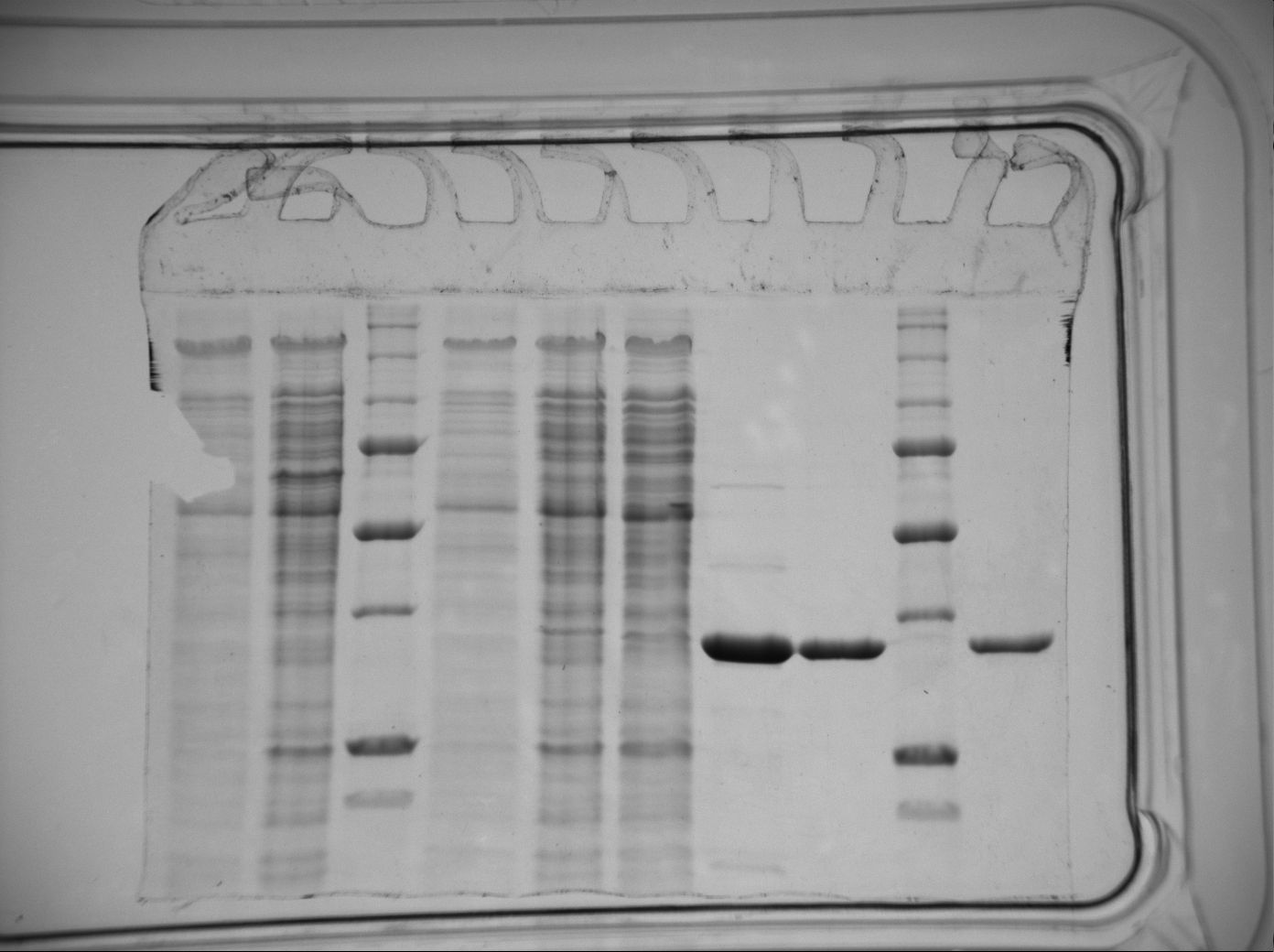


32 kDa

75

50

37

kDa

1

2

25

**Supplemental Figure S2** Rate dependence of xylose isomerase from *H. utahensis* on the D-xylose concentration.

**Supplemental Figure S3** Transcriptional analyses of genes involved in L-arabinose degradation from *H. utahensis*. Northern blotting of Huta_1154 and Huta_1150 was performed with RNA from cells grown on D-glucose (G), D-xylose (X), L-arabinose (A) and D-ribose (R). 16S rRNA served as loading control.

**Supplemental Figure S4** Purified ribokinase from *Halorhabdus timatea*. Coomassie blue stained SDS-PAGE. Lane: 1, molecular mass standard; 2, after Ni-NTA column; 3, after Superdex.


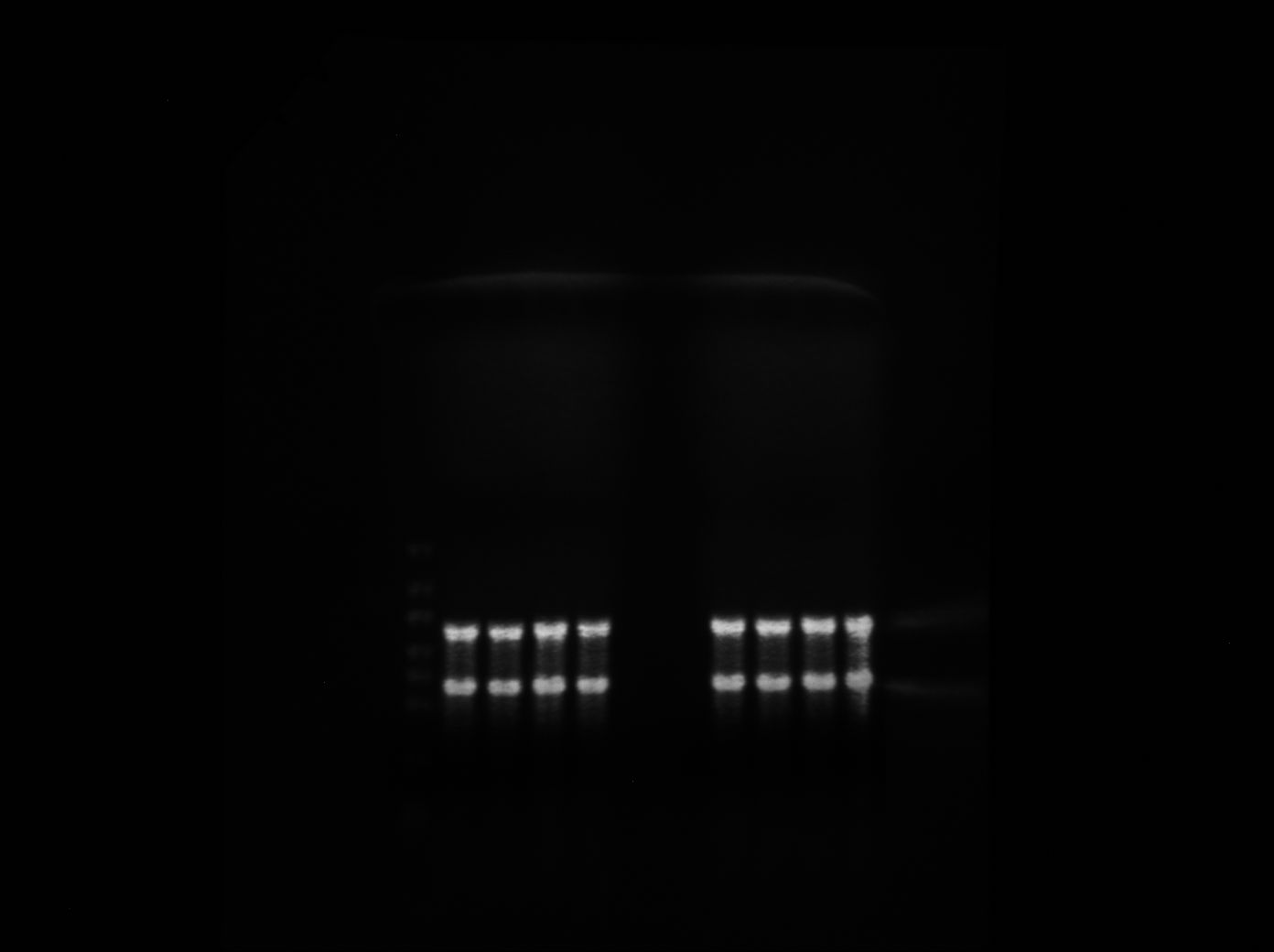

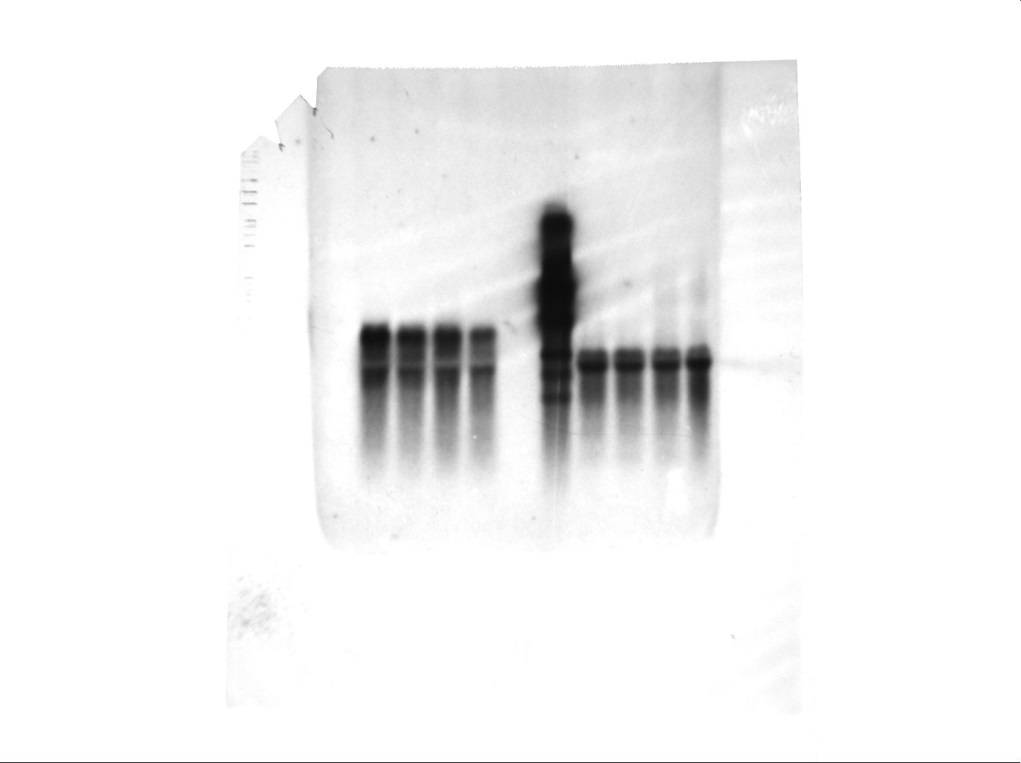


G X A R

2000 nt

1500 nt

1000 nt

Huta_1150

16S rRNA


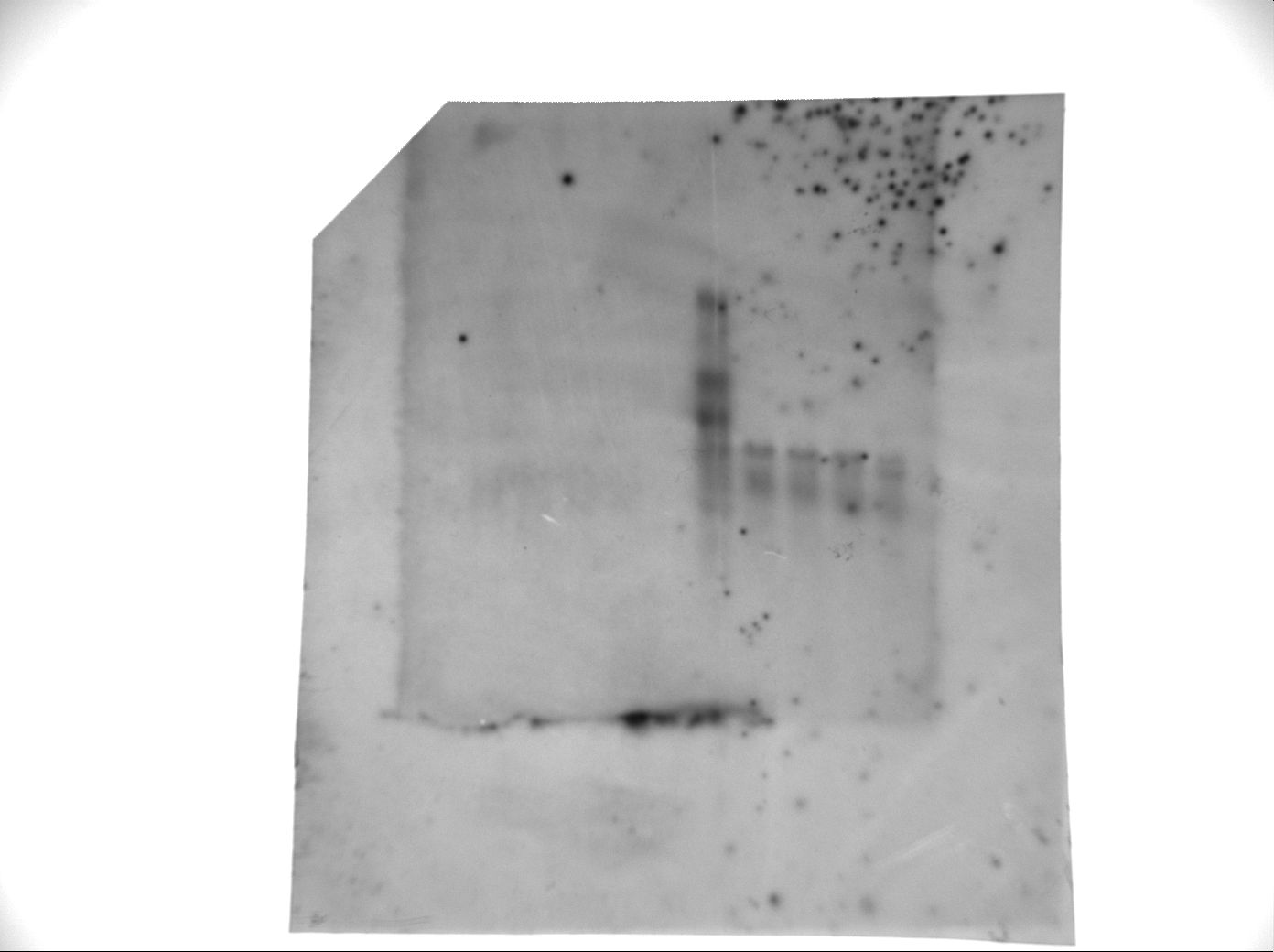


G X A R

2000 nt

1500 nt

1000 nt

Huta_1154


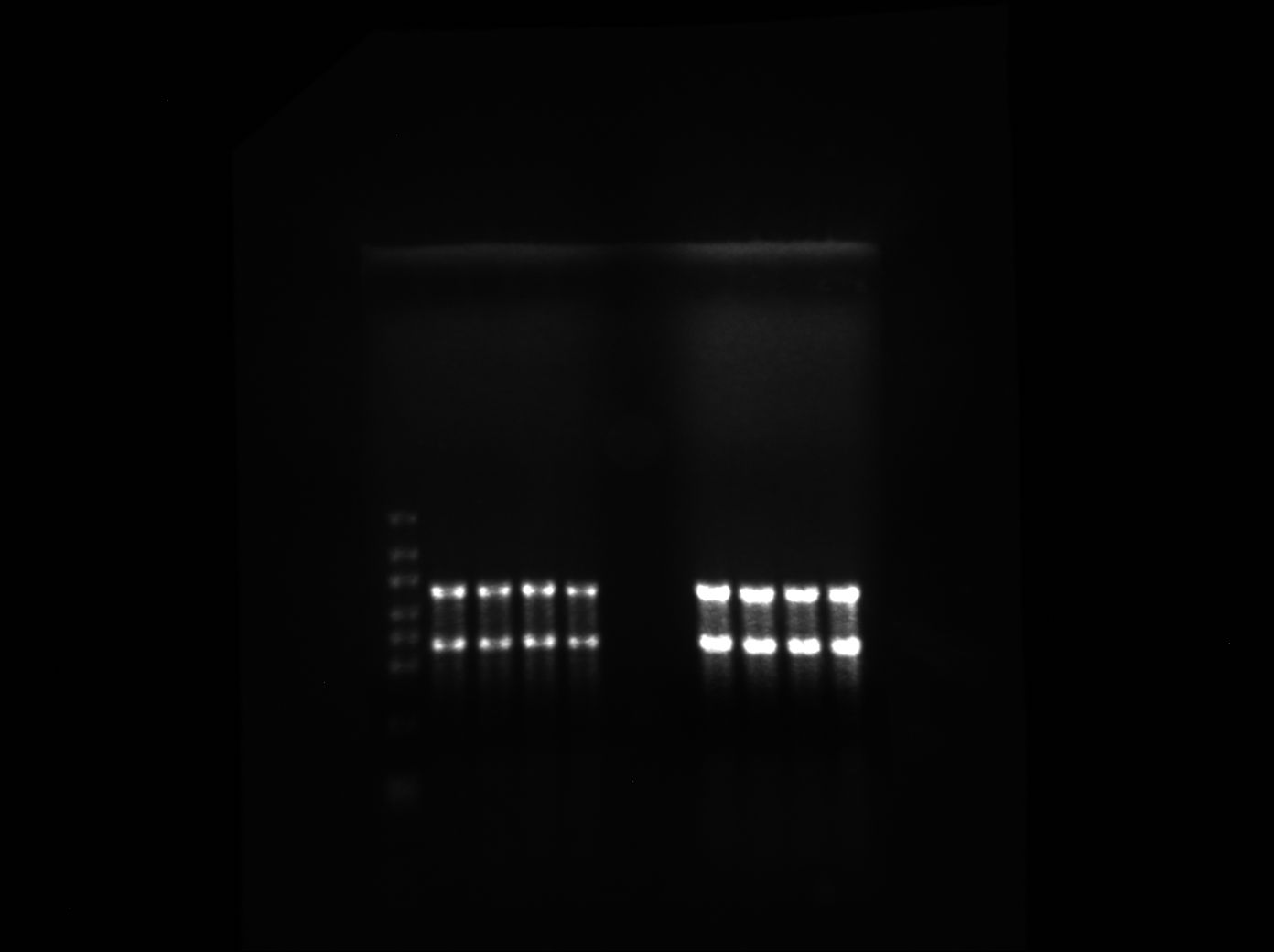


16S rRNA


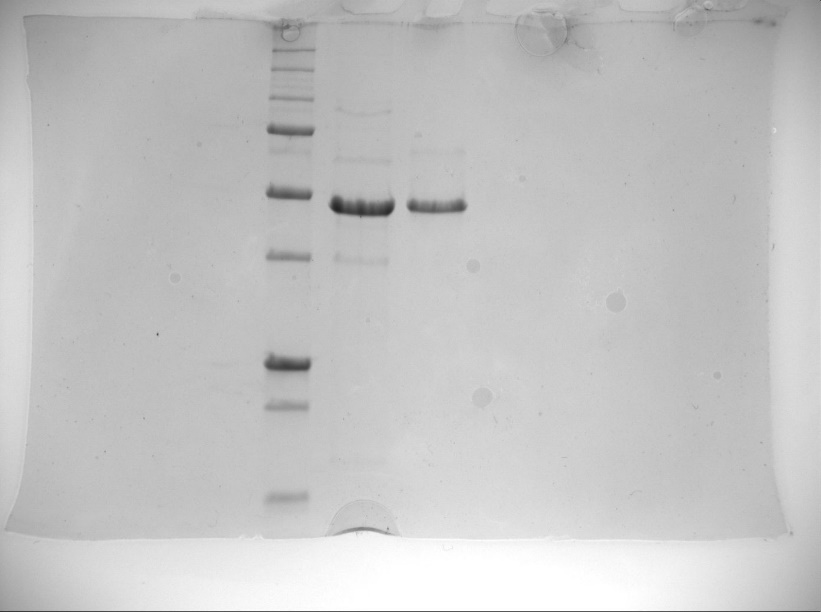


48 kDa

75

50

37

kDa

1 2 3

25

**Supplemental Figure S5** Purification of L-ribulokinase showing ribokinase activity from *H. utahensis* cells grown on D-ribose. (A) Purification as analyzed by SDS-PAGE, Coomassie blue stained; Lanes: 1, molecular mass standard; 2, after Phenyl-Sepharose; 3, after Superdex 200; 4, after Q-Sepharose; (B) Enzyme activitiy was measured at 37°C as ATP-dependent phosphorylation of D-ribose.

A

75

50

37

kDa

1

2

3

4

25

75 kDa


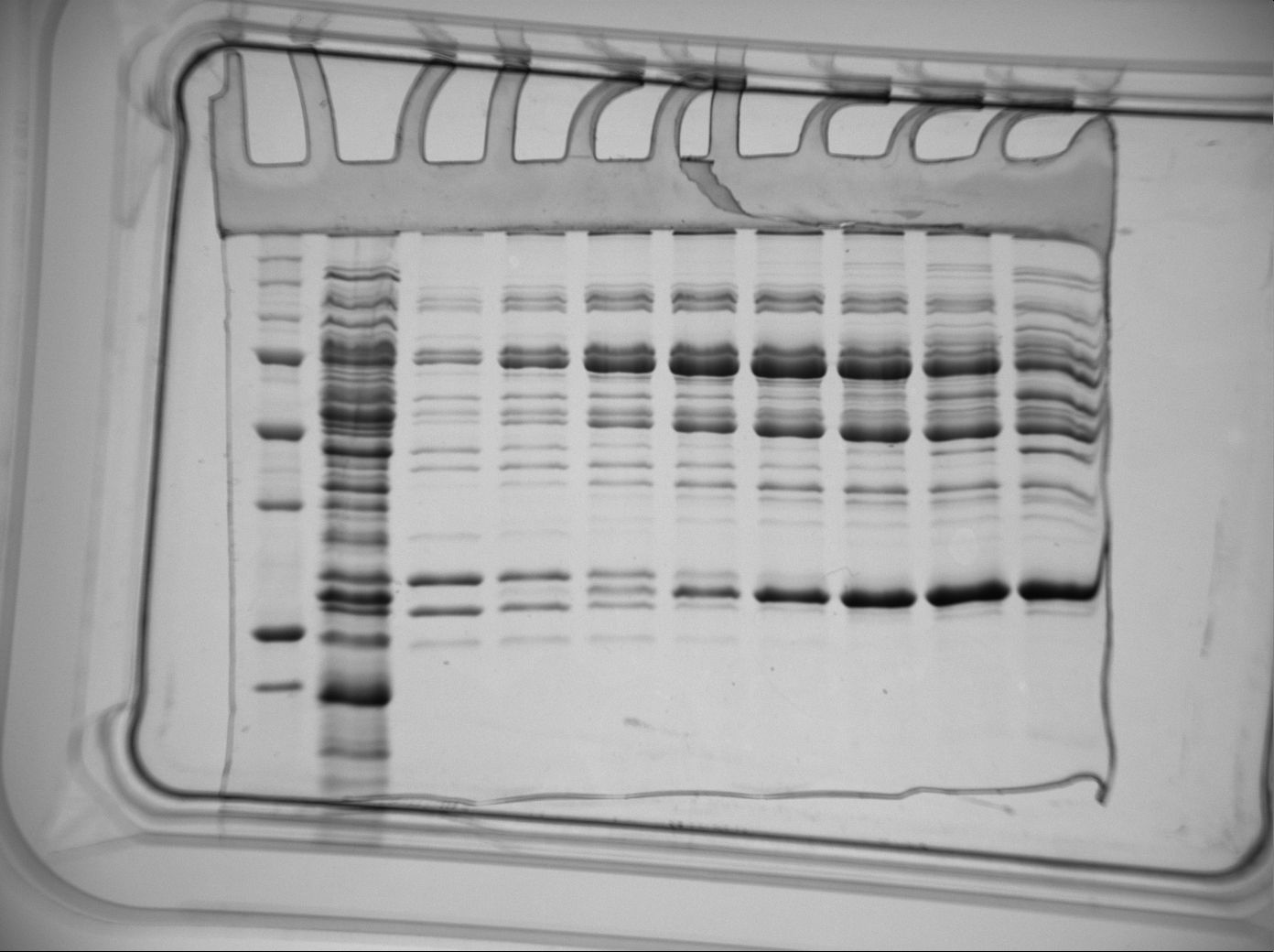

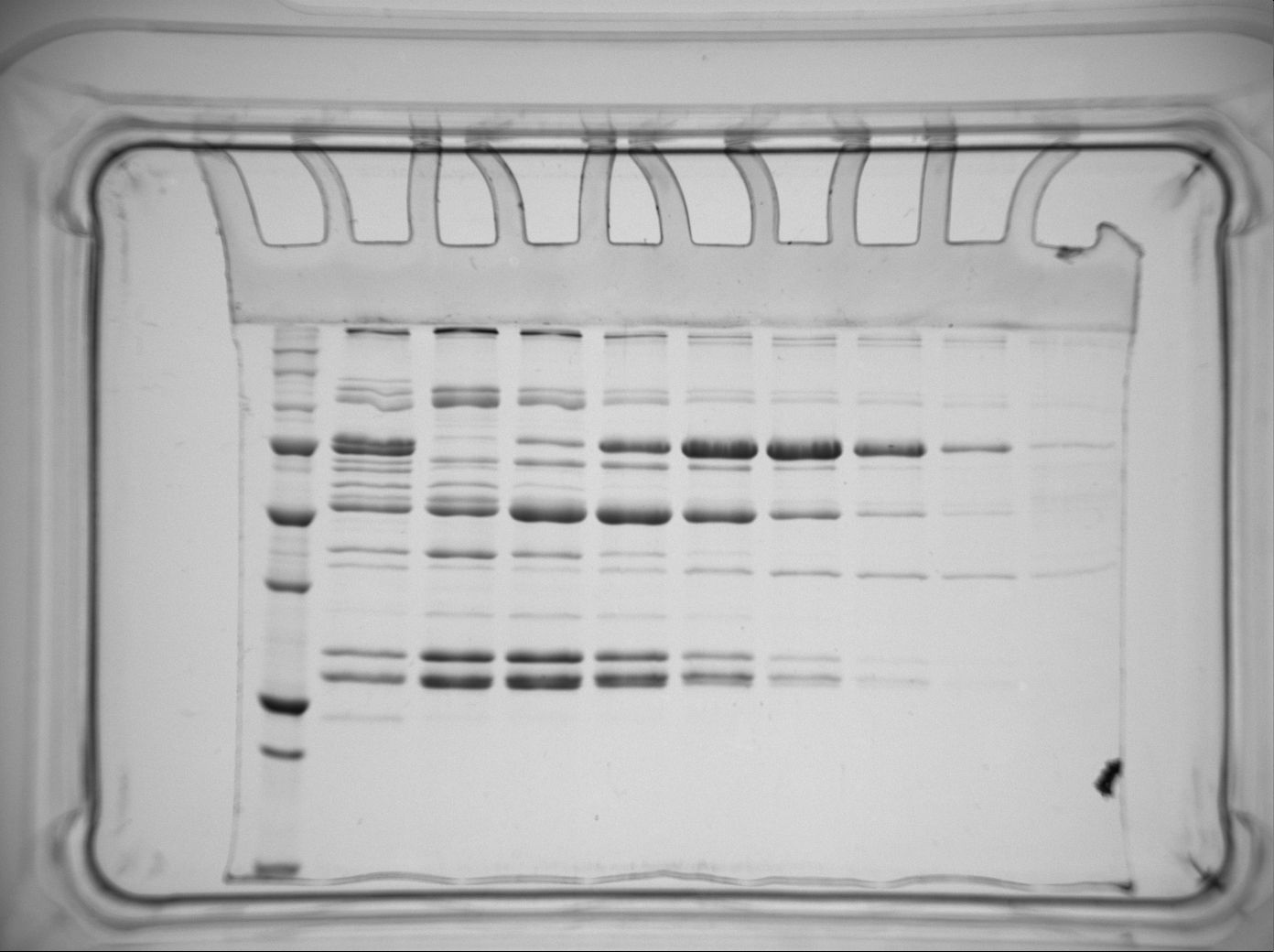

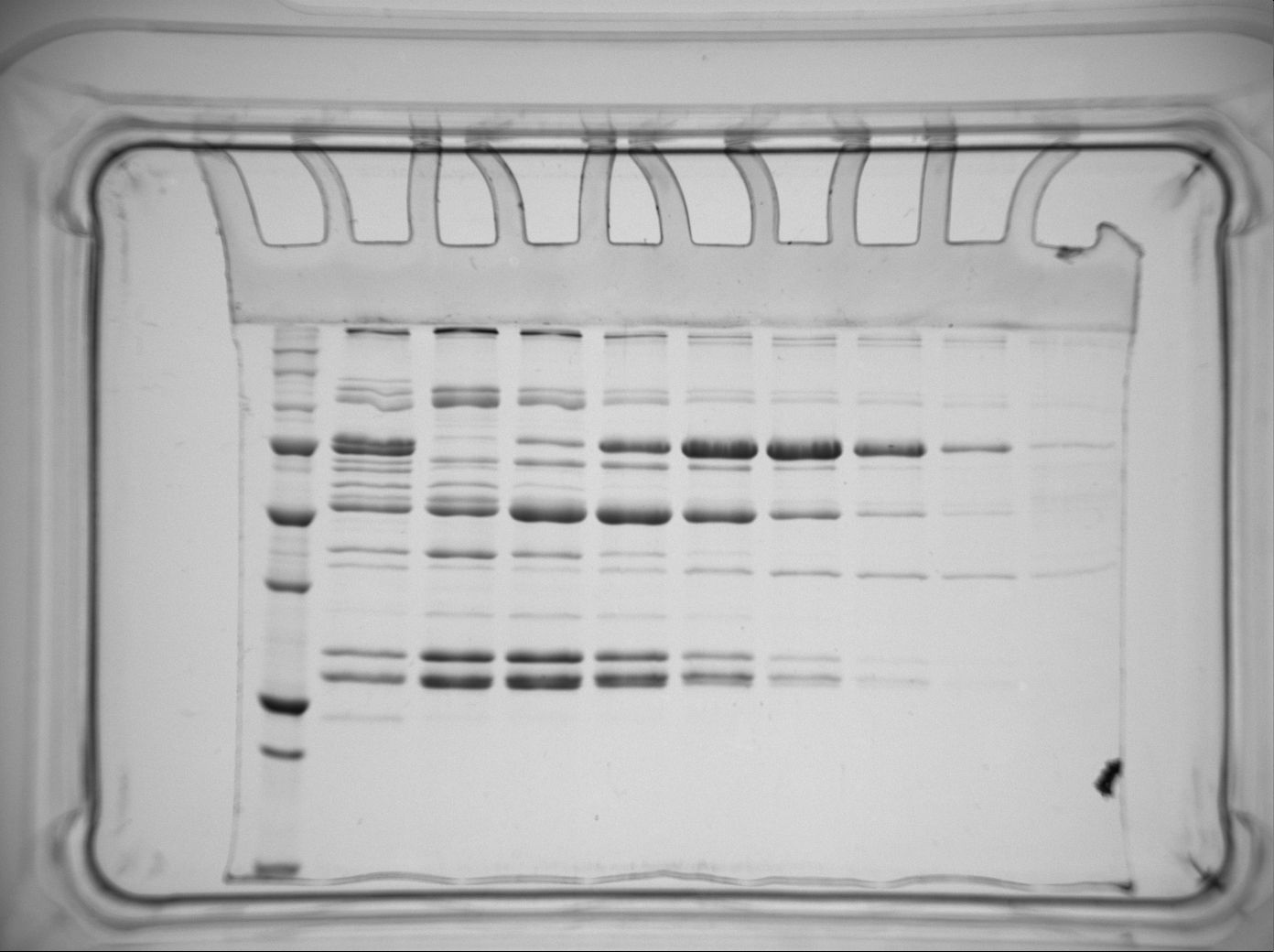


B

|  | **Protein (mg)** | **Activity (U)** | **Specific activity (U/mg)** | **Yield (%)** | **x -fold** |
| --- | --- | --- | --- | --- | --- |
| Cell extract | 671 | 0.595 | 0.0009 | 100 | 1 |
| Phenyl-Sepharose | 21.46 | 0.275 | 0.0128 | 46.22 | 14.2 |
| Superdex 26/60 | 0.5 | 0.156 | 0.314 | 26.22 | 348.9 |
| Q-Sepharose | 0.028 | 0.033 | 1.175 | 5.55 | 1305.6 |

**Supplemental Figure S6** Transcriptional analysis of Huta_0832 from *H. utahensis*. Northern blotting was performed with RNA from cells grown on D-glucose (G), D-xylose (X), L-arabinose (A) and D-ribose (R). 16S rRNA served as loading control.


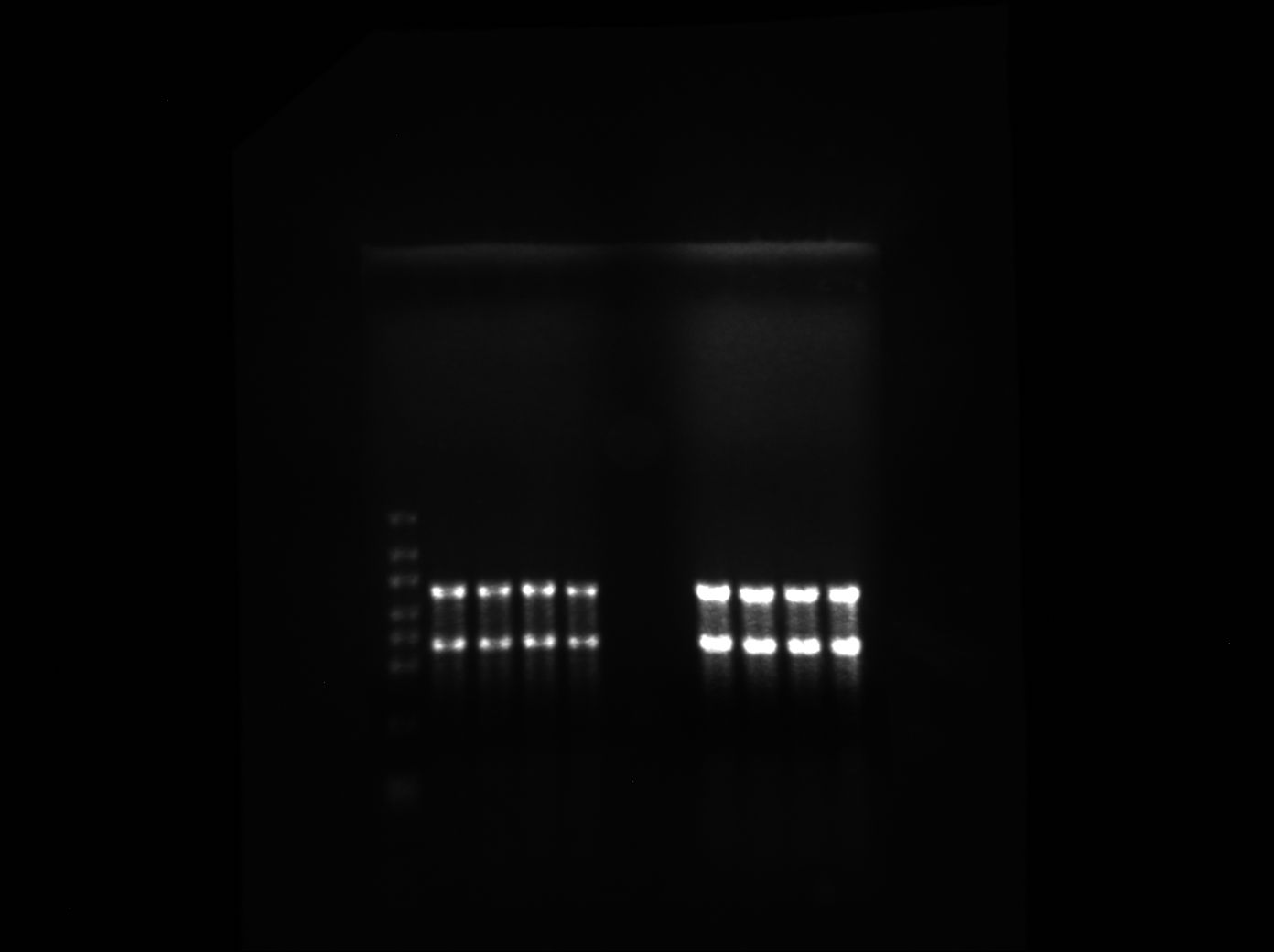

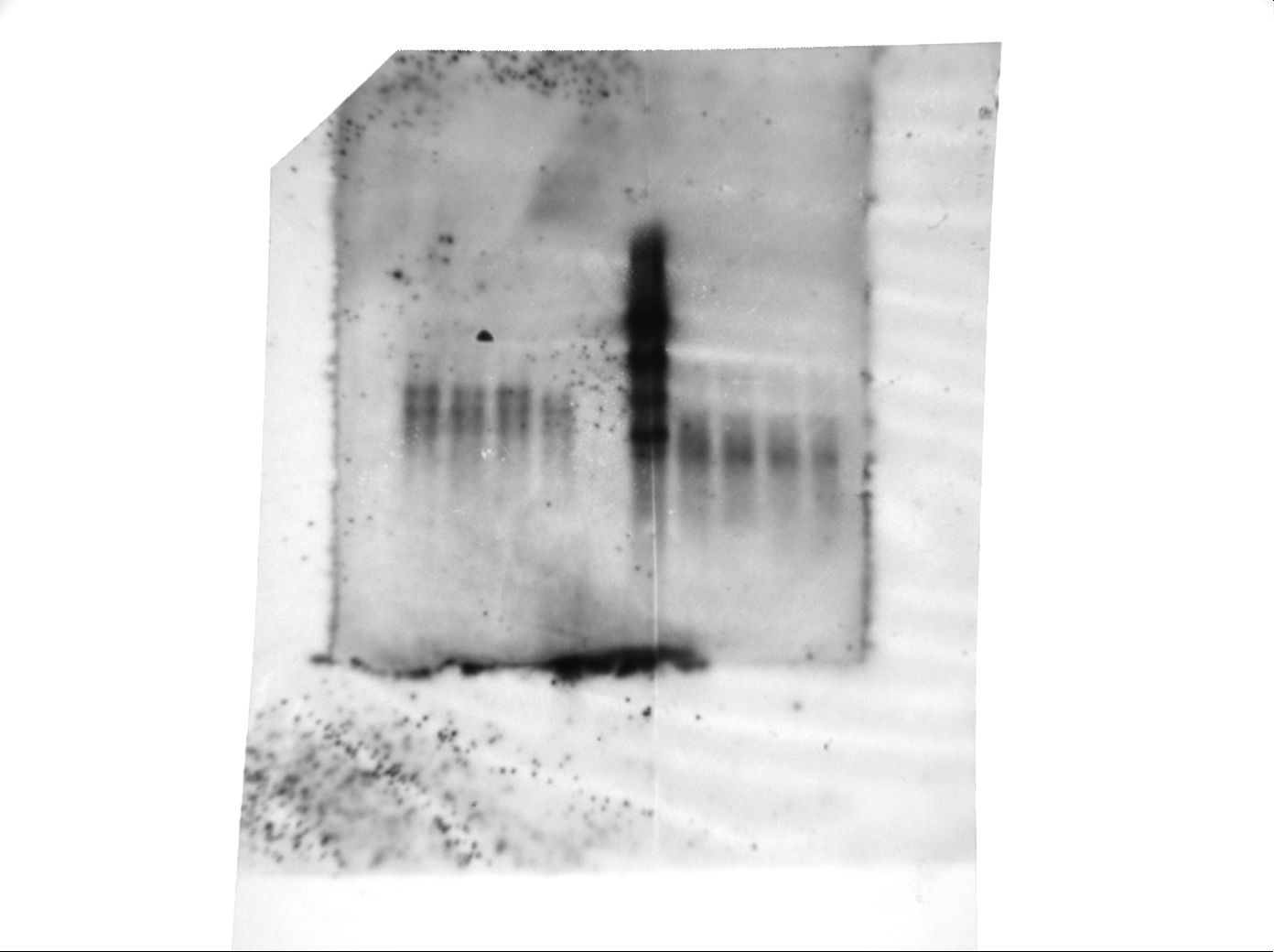


G X A R

2000 nt

1500 nt

1000 nt

Huta_0832

16S rRNA

**Supplemental Table S1** Primers used in this study.

| **Primer name** | **Sequence 5’→ 3’** |
| --- | --- |
| *Primer sequences for cloning genes into pTA963* | |
| Huta_2443BspH1s | gtgatatcatgagcgaatacttcc |
| Huta_2443BamH1as | aggtggagggatcctcactgc |
| Huta_2446NcoIs | actatgagcctttccatggg |
| Huta_2446EcoRIas | gtgccagaattcaacagccg |
| Huta_1150NcoIs | ggagtgattcccatggacggac |
| Huta_1150NotIas | acggcgcggccgcggtgc |
| Huta_1154NdeIs | ttgcgcatatgcaccaccaccaccaccacatgtcacccatcccgcacg |
| Huta_1154NcoIas | accgatgtgaccgccatggagtgacgcg |
| Huta_0832NcoIs | ccaatctcccatggacgaac |
| Huta_0832BamH1as | ctactcggatccggcgtgc |
| HTIA_0439 | Gene was synthesized by Eurofins |
|  |  |
| *Primer sequences for amplification of probes for Northern blotting* | |
| Huta_2443NBs | tcatggagccgaagttcg |
| Huta_2443NBas | attcccgtcgaaccttcg |
| Huta_2446NBs | atccggctgactggtggg |
| Huta_2446NBas | cgaaccccagacgtcacc |
| Huta_1154NBs | tgcatcggcctcatcacc |
| Huta_1154NBas | tccatcagcgatgtgccg |
| Huta_1150NBs | gcatctccagcctctacg |
| Huta_1150NBas | tcttttcctcgccctcgg |
| Huta_0832NBs | cgaagcgacgggcaagcg |
| Huta_0832NBas | acgccgtcctcagtcccc |
